# No Association between HIV and Intimate Partner Violence among Women in 10 Developing Countries

**DOI:** 10.1371/journal.pone.0014257

**Published:** 2010-12-08

**Authors:** Guy Harling, Wezi Msisha, S. V. Subramanian

**Affiliations:** 1 Department of Society, Human Development and Health, Harvard School of Public Health, Boston, Massachusetts, United States of America; 2 Europe and Central Asia Human Development Sector, The World Bank, Washington, D.C., United States of America; Stanford University, United States of America

## Abstract

**Background:**

Intimate Partner Violence (IPV) has been reported to be a determinant of women's risk for HIV. We examined the relationship between women's self-reported experiences of IPV in their most recent relationship and their laboratory-confirmed HIV serostatus in ten low- to middle-income countries.

**Methodology/Principal Findings:**

Data for the study came from the most recent Demographic and Health Surveys conducted in Dominican Republic, Haiti, India, Kenya, Liberia, Malawi, Mali, Rwanda, Zambia and Zimbabwe. Each survey population was a cross-sectional sample of women aged 15–49 years. Information on IPV was obtained by a face-to-face interview with the mother with an 81.1% response rate; information on HIV serostatus was obtained from blood samples with an 85.3% response rate. Demographic and socioeconomic variables were considered as potentially confounding covariates. Logistic regression models accounting for multi-stage survey design were estimated individually for each country and as a pooled total with country fixed effects (n = 60,114). Country-specific adjusted odds ratios (OR) for physical or sexual IPV compared to neither ranged from 0.45 [95% confidence interval (CI): 0.23–0.90] in Haiti to 1.35 [95% CI: 0.95–1.90] in India; the pooled association was 1.03 [95% CI: 0.94–1.13]. Country-specific adjusted ORs for physical and sexual IPV compared to no sexual IPV ranged from 0.41 [95% CI: 0.12–1.36] in Haiti to 1.41 [95% CI: 0.26–7.77] in Mali; the pooled association was 1.05 [95% CI: 0.90–1.22].

**Conclusions:**

IPV and HIV were not found to be consistently associated amongst ever-married women in national population samples in these lower income countries, suggesting that IPV is not consistently associated with HIV prevalence worldwide. More research is needed to understand the circumstances in which IPV and HIV are and are not associated with one another.

## Introduction

Violence against women has been identified as a major risk factor for HIV infection among women.[1], [2], [3], [4], [5] Studies have shown that a woman's exposure to violence, mainly intimate partner violence (IPV), is associated observationally with an increased risk for HIV infection in India,[6] and southern and eastern Africa.[7], [8], [9], [10], [11], [12], [13], [14] In addition, two cohort studies in South Africa have found reduced HIV risk behaviors[13] and reduced HIV incidence[15] following interventions to empower women. There are many mechanisms through which increased IPV could be related to increased risk for HIV infection among women. These include direct effects through higher levels of violent sexual intercourse and indirect effects through reduced ability to negotiate condom use and through an increased likelihood that women who have suffered past abuse are also likely to have more partners, more transactional sex, be less likely to test/disclose and may be less receptive to HIV awareness programs, reflecting an underlying power imbalance.[1], [4], [5], [16], [17] This is in addition to the likelihood that men who enact IPV will have more high-risk sexual behaviors,[18], [19], [20], [21], [22] and may be more likely to be HIV infected in the first place.[23]

Given the plausibility of a positive association between IPV and HIV among women, it is important to evaluate whether such an association is present across multiple country contexts using large, population-based samples. We test the hypothesis that women's self report of IPV is associated with their risk of being infected with HIV in the 10 lower income countries across three continents for which nationwide data on HIV and IPV are currently available.[24]

## Methods

### Data Source

The data for this study came from Demographic and Health Surveys (DHS) conducted in 10 countries between 2003 and 2007: Dominican Republic, Haiti, India, Kenya, Liberia, Malawi, Mali, Rwanda, Zambia and Zimbabwe. While DHS surveys have been conducted in more than 80 countries, only in these 10 countries were the same women both asked about their experiences of IPV and tested for HIV. The target population in each survey that included information on IPV and HIV was men and women in the age range of 15 to 49 years.

### Sampling Plan

The DHS employs a multistage stratified design with probabilistic sampling with each household having an equal probability of selection. Every survey was stratified by urban and rural status and additionally by country-specific geographic or administrative regions. Detailed sampling plans are available from survey final reports.[25], [26], [27], [28], [29], [30], [31], [32], [33], [34] In **Table S1** we describe each survey along with sampling characteristics, response rates and eligible sample sizes. Of a total of 244,004 women eligible for the main questionnaires across the ten surveys, 94.9% participated.

### Study population and sample size

The study population consisted of women aged 15–49 years (n = 231,564). In each country, HIV testing and questions relating to IPV were requested from two independent and randomly selected subsets of the DHS main questionnaire population (**Table S1**).

HIV tests were offered to 140,837 women, of whom 20,745 (14.7% of the HIV-eligible sample) declined the test or their test result was unavailable. IPV questions, as part of the domestic violence (DV) module, were offered to 145,042 women, of whom 27,375 (19.9% of the DV module-eligible sample) did not respond. A total of 60,795 women responded to both the IPV and HIV test questions, from which we excluded those who had missing information on the covariates included in this analysis (n = 681, 1.1%). The final analytic sample for the pooled analysis was 60,114. Since the DV module was only asked of women who were then or had ever been married, this analysis is of ever-married women only.

### Outcome

The outcome was a dichotomous variable indicating HIV serostatus for each woman. Serostatus was determined by collecting dried blood spot samples from each individual. The samples were laboratory tested in serial using two different enzyme-linked immunosorbent assay (ELISA) tests. Any discordant samples were then subjected to a confirmatory Western Blot test. Details of the tests used have been described elsewhere.[35]

### Exposure

IPV was evaluated using questions in the DV module covering two domains of possible abuse by a woman's husband or partner: physical and sexual.

#### Physical

Each survey asked respondents about whether the respondent's most recent partner had: slapped her; twisted her arm or pulled her hair; pushed or shaken her, or thrown something at her; punched her with his fist or with something that could hurt her; try to choke or burn her on purpose; threatened or attacked her with a weapon. Some surveys combined some of these questions, and the Haiti DHS did not ask questions relating to weapons (**Table S2**). A single binary measures were created for these five questions (four for Haiti) with a value of one if a positive response (‘often’ or ‘sometimes’) was given to any of the actions listed, zero otherwise.

#### Sexual

Respondents were also asked whether the respondent's most recent partner had: physically forced her to have sexual intercourse with him; forced her to perform any other sexual acts. A binary measure was created with a value of one if the woman responded to either question in the affirmative, zero otherwise.

### Covariates

We included covariates that have been considered in previous studies examining the association between IPV and HIV among women. These included each woman's age, marital status, education, occupation, religion and lifetime number of sexual partners, and their household's wealth and urban/rural status. Household wealth was defined in terms of ownership of material possessions, with each woman assigned a wealth score based on a combination of 33 different household characteristics that were weighted according to a factor analysis procedure.[36]

### Analysis

We estimated the odds ratios (OR) of each IPV outcome measure using logistic regression models that took account of the survey design of the studies by allowing for clustering at the level of the primary sampling unit (typically a village or census area). We did not use the sample weights provided by DHS for the full samples of HIV test and DV module participants, since our samples included only the subgroup of individuals who responded to both sets of questions, and thus it is unclear what population such weights would lead our results to be representative of. Models were fitted using Stata v11.0 (StataCorp, College Station, TX). Statistical precision was ascertained using two-tailed Wald tests and results are presented with 95% confidence intervals (CI).

We first estimated the unadjusted association between reported IPV and HIV, both for each national sample separately and pooled across all countries. For pooled analyses country-level fixed effects for each country were included each model. We then re-estimated this association including other covariates which were believed to have affected both a woman's likelihood of reporting IPV and of being HIV positive. We first compared women who reported either physical or sexual IPV to those reporting neither. We then focused on those women reporting sexual IPV, dividing them into those reporting sexual but not physical IPV and those reporting both, and comparing each group to those reporting no sexual IPV using a multinomial model.

To test the robustness of the observed findings, we conducted several sensitivity tests. First, for six countries information was available on who refused to be tested for HIV we conducted two-sample tests for the equality of proportions of women who did and did not refuse HIV tests, for each measure of IPV. Second, we re-estimated our models using the two sets of sample weights provided by the DHS (one for HIV test-eligible women; the other for DV module-eligible women) both individually and jointly to see if the results found were affected by the choice of weights used. Third, we estimated the pooled models without India, which comprised 49.4% of the total sample size, to test whether the pooled results were being driven by a single country. Fourth, we considered whether the pooled analysis was overly simplistic in not considering effect modification of the IPV-HIV relationship by country, by including interaction terms between the IPV exposure measures and country fixed effects; we tested for homogeneity of IPV -country effects using a Wald-type F-test for the joint probability of no interaction. Fifth, we estimated each regression separately for currently and previously married women, to determine whether the IPV-HIV relationship was different in these two populations.

### Ethical Review

Each DHS survey was conducted under the scientific and administrative supervision of a local country organization and was reviewed by the relevant ethics review board. Data collection procedures were also approved by the ORC Macro institutional review board. Informed consent was gained for the survey and for HIV testing.[35] This study was reviewed by Harvard School of Public Health Institutional Review Board and was considered exempt from full review as it was based on an anonymous public use data set with no identifiable information on survey participants.

## Results

The overall prevalence of HIV among women in this study was 4.3% (**Table 1**; detailed results for each country in **Table S3**); the rate was highest in Zimbabwe (24.1%) and lowest in India (0.5%). Almost one-third (32.1%) of women surveyed reported having experienced some form of IPV in their most recent sexual relationship. Almost one-third of women reported experiencing physical IPV (30.0%) with their most recent partner, one in eleven reported experiencing sexual IPV (8.6%), and 6.5% of women reported experiencing both. The country-specific adjusted rates of physical and sexual IPV were strongly correlated with one another (ρ = 0.649; p = 0.04). The distribution of the covariates across countries overlapped substantially and there was evidence for demographic and socioeconomic patterning of IPV (pooled results shown in **Table 2**; detailed results for each country in **Table S4**).

**Table 1:** HIV prevalence in each country sample by type of intimate partner violence exposure.

		Total sample			Any Physical IPV			Physical or sexual IPV			Any Sexual IPV			Physical and sexual IPV		
Sample	Survey year	Sample size	HIV-positive	%	Exposed	%	% exposed HIV-positive	Exposed	%	% exposed HIV-positive	Exposed	%	% exposed HIV-positive	Exposed	%	% exposed HIV-positive
Pooled	-	60,114	2,597	4.3	18,011	30.0	5.0	19,296	32.1	5.1	5,197	8.6	7.1	3,912	6.5	6.9
Dominican Republic	2007	7,870	86	1.1	1,238	15.7	1.5	1,308	16.6	1.5	435	5.5	1.4	365	4.6	1.6
Haiti	2006	2,628	96	3.7	312	11.9	2.6	457	17.4	2.2	278	10.6	1.8	133	5.1	2.3
India	2005/06	29,783	144	0.5	10,170	34.1	0.6	10,498	35.2	0.7	2,057	6.9	0.9	1,729	5.8	0.9
Kenya	2003	1,756	153	8.7	700	39.9	8.6	757	43.1	8.6	252	14.4	6.7	195	11.0	6.2
Liberia	2007	3,278	80	2.4	1,177	35.9	2.5	1,268	38.7	2.4	302	9.2	2.0	211	6.4	1.9
Mali	2006	2,804	47	1.7	453	16.2	2.0	482	17.2	2.1	99	3.5	3.0	70	2.5	2.9
Malawi	2004	2,086	327	15.7	451	21.6	17.1	588	28.2	16.7	291	14.0	15.8	154	7.4	16.2
Rwanda	2005	2,476	81	3.3	770	31.1	3.6	869	35.1	3.7	344	13.9	4.7	245	9.9	4.9
Zambia	2007	3,368	603	17.9	1,514	45.0	19.2	1,433	35.3	24.4	573	17.0	21.3	451	13.0	23.1
Zimbabwe	2005/06	4,065	980	24.1	1,226	30.2	25.2	1,636	48.6	18.8	566	13.9	22.6	359	8.8	24.5

Note. Each item of intimate partner violence is a binary measure of those women reporting any experiences in the category.

**Table 2:** Pooled sample size and percentage distribution by exposure and covariates.

	N	%	% HIV-positive
**Age**			
15–19	3,286	5.5	2.8
20–24	10,367	17.2	4.1
25–29	12,833	21.3	5.0
30–34	11,975	19.9	5.2
35–39	9,380	15.6	4.3
40–44	6,972	11.6	3.7
45–49	5,301	8.8	3.0
**Marital status**			
Currently	54,203	90.2	3.5
Formerly	5,911	9.8	11.8
**Urbanity**			
Urban	24,939	41.5	4.1
Rural	35,175	58.5	4.5
**Wealth quintiles**			
Poorest	10,644	17.7	4.0
2^nd^ poorest	11,520	19.2	3.8
Middle	12,504	20.8	4.1
2^nd^ richest	13,371	22.2	5.3
Richest	12,075	20.1	4.2
**Education**			
None	19,401	32.3	1.9
Primary	18,192	30.3	6.7
Secondary & above	22,521	37.5	4.4
**Occupation**			
Not employed	28,732	47.8	4.0
Agricultural	14,945	24.7 9	3.8
Manual	4,608	7.7	2.8
Non-manual, non-agricultural	11,829	19.9 7	6.3 4
**Religion**			
Christian	19,297	36.9	10.8
Muslim	7,253	13.9	1.9
Hindu	23,321	44.6	0.5
Other/none	2,373	4.5	6.7
**Lifetime number of partners**			
Zero or one	42,730	75.9	1.9
Two or more	13,542	24.1	9.6
**Intimate partner violence**			
No physical nor sexual violence	40,818	67.9	3.9
Any physical or sexual violence	19,296	32.1	5.1
Any physical violence	18,011	30.0	5.0
Any sexual violence	5,197	8.6	7.1
Physical and sexual violence	3,912	6.5	6.9

**Figure 1:**
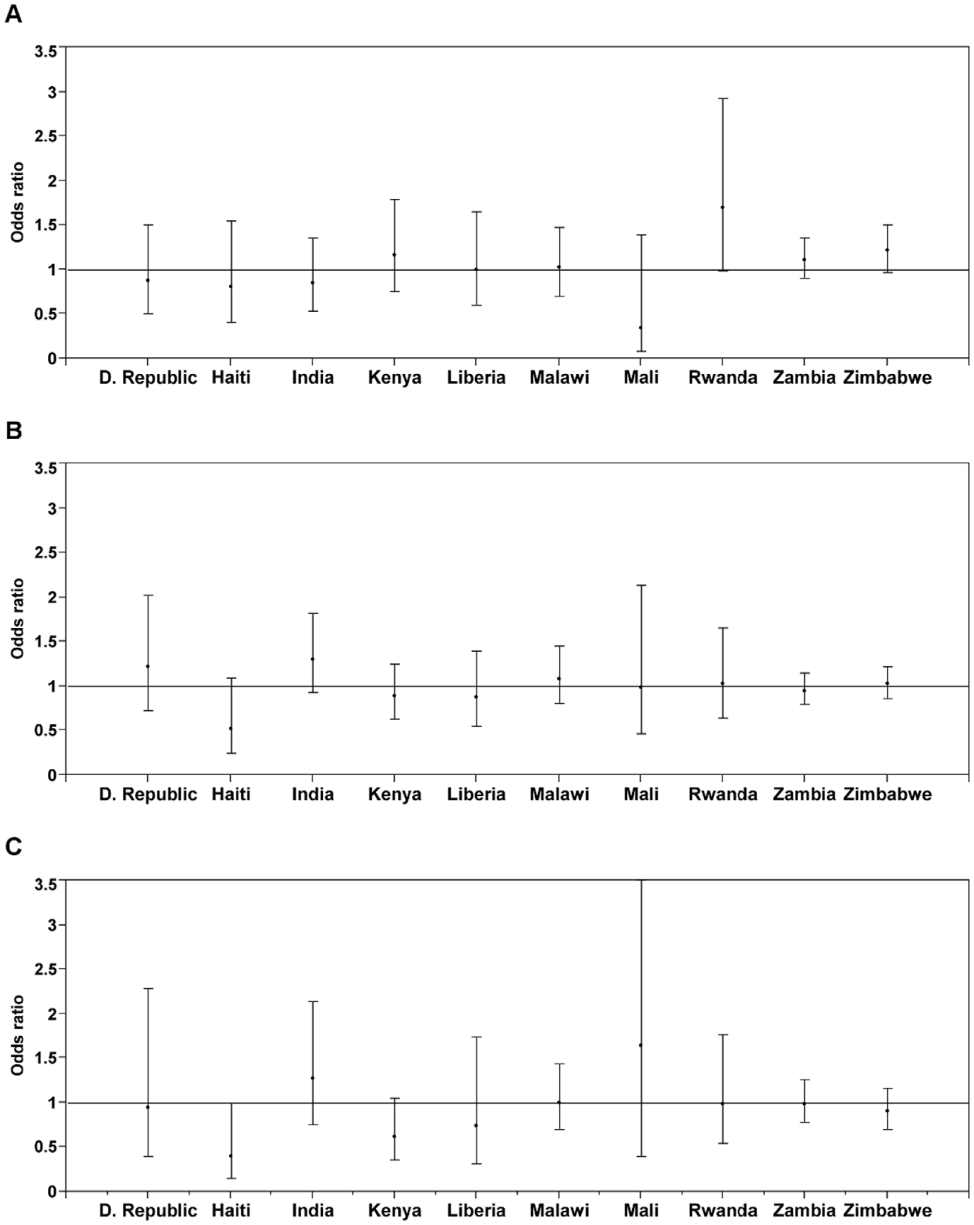
Adjusted associations between HIV and self-reported intimate partner violence. Each association includes a point estimate and 95% confidence interval: A. Any physical or sexual IPV vs. neither form of IPV, B. Sexual but no physical IPV vs. no sexual IPV, C. Both physical and sexual violence vs. no sexual IPV.

In the pooled unadjusted regression analysis, a significant positive association between different categories of IPV experienced and HIV infection was observed, except for ‘sexual without physical violence’ (**Table 3**). The unadjusted odds ratios for HIV infection was strongest when comparing those who had experienced both physical and sexual IPV to those who had experienced no sexual IPV (OR 1.20; 95% CI 1.04, 1.39) and weakest when comparing those who had experienced sexual but not physical IPV to those who had experienced no sexual IPV (OR 1.06; 95% CI 0.97, 1.17). However, adjustment for covariates attenuated the point estimates for each of the IPV domains (**Figure 1**; detailed results of all covariate coefficient estimates in **Table S5**). In country-specific unadjusted analyses, Indian women who experienced any combination of IPV appeared to be at increased risk for HIV infection compared to those who did not, while Haitian women who reported experiencing either physical or sexual IPV were at lower risk of HIV infection in the adjusted analyses. In no other of the ten countries was there a statistically significant association between any combination of reported IPV and HIV before or after covariate adjustment.

**Table 3:** Unadjusted and adjusted odds ratios [95% confidence intervals] for the association between HIV prevalence and intimate partner violence.

			Physical or sexual violence vs. neither		Sexual without physical violence vs. no sexual violence		Physical & sexual violence vs. no sexual violence	
Sample	Sample size	# HIV positive	Unadjusted	Adjusted	Unadjusted	Adjusted	Unadjusted	Adjusted
Pooled *	60,114	2,597	1.10	1.03	1.06	1.02	1.20	1.05
			[1.01–1.19]	[0.94–1.13]	[0.97–1.17]	[0.93–1.13]	[1.04–1.39]	[0.90–1.22]
Dominican Republic	7,870	86	1.43	1.12	1.36	1.11	1.62	1.14
			[0.87–2.36]	[0.67–1.88]	[0.75–2.44]	[0.62–2.00]	[0.70–3.74]	[0.47–2.78]
Haiti	2,628	96	0.54	0.45	0.54	0.48	0.56	0.41
			[0.28–1.05]	[0.23–0.90]	[0.25–1.16]	[0.22–1.04]	[0.17–1.81]	[0.12–1.36]
India	29,783	144	1.69	1.35	1.59	1.35	2.24	1.34
			[1.23–2.34]	[0.95–1.90]	[1.13–2.24]	[0.94–1.94]	[1.29–3.89]	[0.73–2.44]
Kenya	1,756	153	0.97	0.88	1.08	1.01	0.68	0.54
			[0.69–1.37]	[0.62–1.25]	[0.76–1.53]	[0.70–1.44]	[0.34–1.35]	[0.27–1.06]
Liberia	3,278	80	1.00	0.87	1.05	0.90	0.77	0.68
			[0.64–1.57]	[0.56–1.35]	[0.63–1.74]	[0.55–1.48]	[0.27–2.24]	[0.24–1.93]
Mali	2,804	47	1.31	1.07	1.22	1.01	1.82	1.41
			[0.68–2.53]	[0.51–2.23]	[0.60–2.49]	[0.48–2.12]	[0.39–8.36]	[0.26–7.77]
Malawi	2,086	327	1.11	1.07	1.12	1.09	1.07	1.01
			[0.85–1.45]	[0.81–1.42]	[0.84–1.50]	[0.80–1.47]	[0.67–1.72]	[0.62–1.65]
Rwanda	2,476	81	1.22	0.99	1.05	0.97	1.64	1.04
			[0.76–1.95]	[0.59–1.67]	[0.56–1.97]	[0.48–1.92]	[0.90–2.98]	[0.56–1.93]
Zambia	3,368	603	1.13	0.91	1.01	0.87	1.46	1.01
			[0.98–1.31]	[0.77–1.08]	[0.86–1.20]	[0.72–1.06]	[1.16–1.83]	[0.77–1.33]
Zimbabwe	4,065	980	1.02	0.97	1.02	0.98	1.03	0.97
			[0.88–1.19]	[0.83–1.15]	[0.86–1.21]	[0.82–1.16]	[0.79–1.35]	[0.72–1.31]

Note: Data are adjusted for clustering but not weighted for sampling probabilities. Odds ratios in country-specific regressions are adjusted for age, marital status, urbanity, household wealth, education, occupation, religion and lifetime number of partners, except for the Dominican Republic (no religion) and Kenya and Malawi (no lifetime number of partners). Odds ratios in the pooled regression are adjusted for age, marital status, urbanity, household wealth, education and occupation.

*Country-level fixed effects are included for the pooled regression, but values are not shown in this table.

Rerunning the analyses weighted either by the DV sample weights, the HIV sample weights or a combination of the two did not significantly affect the overall pooled results, although the results for individual countries did change in some cases (**Table S6**). Similarly, rerunning the pooled analyses excluding India made little difference; both the unadjusted and adjusted ORs were generally slightly lower than for all ten countries together (**Table S7**). Comparing the results specifically for India, by different forms of IPV, adjustments and weightings, the associations were similar for comparisons of women who had experienced physical or sexual, or sexual but not physical IPV, but were consistently larger for sexual and physical violence (**Table S7**).

Tests of homogeneity for each of the six pooled models (adjusted and unadjusted for each of the three outcomes) when rerun to test for variation in the IPV-HIV relationship across countries were marginally able to reject homogeneity in one case (adjusted analysis for physical or sexual IPV vs. no IPV: χ^2^ = 1.90, p-value = 0.048), but unable to reject the null in the other five cases (not shown). It therefore appears that heterogeneity of effects was limited in this study. Separate pooled analyses for currently and previously married women showed that previously married women had slightly lower odds ratios for the IPV and HIV association, but no significant changes in the results were found (**Table S8**).

## Discussion

Using all currently available DHS surveys, based on laboratory tests of HIV and self reports of IPV we find no robust or consistent association between reported physical and sexual IPV and HIV infection amongst women in ten national population samples. In the pooled data, while unadjusted relationships found IPV to be associated with a small, significant increase in HIV prevalence (OR between 1.06 and 1.20), these relationships attenuated into insignificance once adjustment was made for demographic and social factors (OR between 1.02 and 1.05). This suggests that those experiencing IPV have a higher prevalence of HIV in this dataset, but that this association can be explained by precedent, common risk factors.

Country-specific regressions show that effect sizes were small in almost all cases – adjusted associational ORs were generally between 0.85 and 1.15 for all IPV exposures. The exceptions to this were India (with ORs of between 1.34 and 1.35) and Haiti (ORs between 0.41 and 0.48). Additionally, when comparing those experiencing physical and sexual IPV to those reporting no sexual IPV, Mali mimicked India, and Kenya and Liberia were closer to Haiti. Nevertheless, only one of thirty adjusted country-specific analyses recorded a significant association.

The study's findings should be interpreted with the following caveats. First, the cross-sectional nature of the data is of generic concern when considering any causal connection between variables, severely limiting our ability to ascertain the temporality of any association shown. For instance, it is possible that IPV is a consequence of a woman being HIV-positive. The data used here are stronger than some other cross-sectional data in that most women who tested HIV-positive will be unaware of their status prior to the interview,[37] thus reducing the connection between seropositivity and partner awareness. Second, there remains a possibility of selection bias, if those who participated in HIV testing or the domestic violence module differed from those who did not. Information on those refusing the domestic violence module was not available, however comparisons of those accepting and refusing an HIV test – which found those refusing an HIV test to report either similar or lower IPV rates than those accepting a test (**Table S9**) – suggest that any selection bias introduced would have acted to inflate rather than deflate our results.

Third, the measures of IPV collected by DHS are imperfect. The questions asked relate only to each woman's last “husband/partner”, and do not ask about frequency of IPV except in the categories of ‘often’ or ‘sometimes’. This makes the meaning of any reported association harder to interpret. The meaning of the question may have been systematically different for women who do and do not have a current partner; however our sensitivity analysis stratifying by marital status suggests that the results found are qualitatively similar for married and previously married women. Fourth, in several of the countries the number of women testing positive was small (fewer than 100 in the Dominican Republic, Haiti, Liberia, Mali and Rwanda), which may have reduced the power available to test for a significant association between IPV and HIV, such that a true association might not have been seen. However, in these five countries no adjusted effect size was larger than 1.14. This concern is also somewhat allayed by the results from the pooled regressions, which follow the pattern of the individual country regressions in showing a small, significantly positive relationship in the unadjusted regressions, which is then attenuated in the adjusted analyses. Furthermore, given that country-specific results are equally spread above and below the null value, increased power would not change our overall finding of no clear effect direction.

Fifth, the data analyzed are only for women who are, or have previously been, married or cohabiting with partners (24.7% of all DHS respondent women had never married). Consequently, inferences cannot be generalized to all women. As noted elsewhere [38], there is no consistent patterning of IPV by marital status or age, so it is unclear how this limitation of the sample affects how results can be extrapolated to all women, or compared to the existing literature. Nevertheless, with high lifetime marriage rates in most countries, the population covered includes the majority of the world's female population. Finally, although this study includes countries with a wide range of HIV epidemics – from the hyperendemic to the highly concentrated – reported IPV rates and models of gender-based behavior, the countries with all necessary data were not randomly selected with respect to the world's population. Generalizing from this dataset to other countries will require careful consideration of the state of HIV and IPV in other countries, as well as other normative beliefs and behaviors.

The findings of this analysis provide new evidence in the ongoing effort to understand the relationship between IPV and HIV. The differences between our findings from those previously reported [6], [7], [8], [9], [10], [11], [12], [14], [15] may – with the exception of those for India [6] and South Africa [9], [13], [15] – reflect differences in sampling technique since many of the previous studies were based on samples that did not cover the whole female population, often sampling women attending health facilities [7], [8], [11], [12], [14].

We note that the Indian study, using the same data as this analysis, reported a positive association between HIV and IPV for women who had suffered both physical and sexual IPV. However, our analysis suggests that this finding is not robust to different weightings (neither the DV nor the HIV weights provided by DHS are directly relevant to the subsample of women who were invited both to take an HIV test and complete the DV module), or to the addition of previously-married women to the sample (an additional 1801 women, or 6.0%), or to the inclusion of additional covariates (we added marital status, occupation and urban residence). Each of these three factors partially attenuated the initial association (**Table S10**). While the largest association we found in the Indian sample was for the types of IPV most likely to relate to HIV (experience of both physical and sexual violence compared to experience of no sexual IPV), the association was not significant at the 95% confidence level.

The null findings in this study may be a function of the geographic settings considered. While some of the previous studies have been conducted in India [6], and East African countries covered here [8], [12], [14], the strongest evidence for a relationship between IPV and HIV to date has been found in South Africa [7], [9], [13], [15], which does not have the DHS data to allow its inclusion in this analysis. The likelihood that these differences are due to country coverage is lessened by the fact that in Rwanda and Kenya positive associations were found in STI, antenatal and paediatric clinic attendees [8], [12], [14]. This suggests that the difference in results is more likely to be due to the differences in study design than geography in these two countries, although we cannot parse out the contribution of each elsewhere.

Further research is needed to determine whether the relationship between IPV and HIV in specific countries differs depending on whether the study population is clinic-based or uses a national sample, or whether geographical effect-modification is occurring. If clinic populations prove to have a stronger relationship between IPV and HIV, this argues for a more targeted approach to IPV based in clinical, rather than general, populations. If variation is by national setting, it will be important to first determine which factors (HIV-related, IPV-related or some third factor) are present when IPV and HIV are associated, and then to focus on intervening in such settings.

Although evidence for effect-modification by setting is marginal in this study, the findings of a significantly positive unadjusted association in India, and a significantly negative adjusted relationship in Haiti, raise the possibility that the IPV-HIV relationship in these countries may differ from the other eight countries included. If these effects are in fact causal, this may provide useful indicators for future research. In India, Decker and colleagues have found those men who commit IPV also engage more generally in higher levels of gendered HIV risk behaviors, including sexual infidelity, coercive condom practices and transactional sex[18]. In a society where HIV remains highly concentrated amongst Most At-Risk Populations (MARP), including sex workers [39], this combination of risk behaviors may act to put the partners of IPV perpetrators at raised risk of HIV. Research to determine whether similar patterns of behavior are seen in settings where IPV and HIV have been consistently linked – such as South Africa – but perhaps not seen in other countries in this study sample, would be of interest. In contrast, Haiti is a society where HIV is more broadly experienced, although still with higher levels of infection amongst MARPs [40]. It is also a country where social dislocation and economic stagnation loom large. It may be that in this specific context, those factors which put women at risk of IPV actually reduce the risk of their partner being HIV positive, and thus reduce the woman's risk of becoming HIV positive themselves. It is important to note that changes in HIV risk behavior will only affect HIV risk if HIV is present in a partnership's sexual network. Determining risk factors for the presence of HIV in a social network may help us understand why in some contexts IPV and HIV are associated, but elsewhere they are not.

Existing frameworks linking IPV and HIV posit multiple individual-level risk factors, including increased risk of violent intercourse, lower decision-making power and partners' high-risk sexual behaviors leading them to be more likely to be HIV-positive [1]. This study's results, if proven to be causal, suggest that such frameworks linking IPV and HIV may at a minimum be contingent on context-specific factors, such that these risk factors are not differentially distributed across women experiencing and not experiencing IPV. This does not necessarily undermine the relationship between gender inequity and sexual risk: inequity can increase IPV rates, and thus increase risky behavior, but if inequity does not also change the sexual networks within a setting such that HIV is more likely to be present in either partner, then no overall effect will be seen. Alternatively, it may be that focusing on individual- or relationship-level effects is to miss the point [41]: a structural explanation in which gender inequity is a driver of both increased IPV throughout society and HIV in both IPV and non-IPV experiencing populations in consistent is also consistent with our findings. In this case IPV would not be the mechanism through which gender inequity led to HIV, even though settings with more inequity might be linked to higher HIV rates.

Given the associational nature of these findings, and the various other potential limitations highlighted, any policy recommendations based on these results should be tempered by the need to replicate these results in longitudinal data. However, if the weak association between IPV and HIV suggested by our results prove to be causal, it would argue for focusing HIV prevention funds elsewhere than adult IPV prevention. This would not rule out gender empowerment more broadly as an HIV prevention intervention, or addressing childhood IPV in order to reduce adult risk of HIV infection [42]. These results also say nothing regarding the intrinsic importance of reducing IPV.

In summary, this study presents evidence that there is no consistent association between physical and sexual IPV and HIV among ever-married women in ten countries in the Americas, Africa and Asia, once we adjust for commonly considered confounders of the relationship. Given evidence elsewhere of plausible causal mechanisms between the two phenomena, further investigation is needed to understand in which circumstances the two may or may not be associated, and what mechanisms may be at play in determining the strength of the relationship. The null findings reported here do not negate the importance of reducing IPV as an intrinsically important public health goal and a basic human right, but do suggest that IPV is not consistently associated with HIV prevalence worldwide.

## Supporting Information

Table S1 Sampling characteristics of the ten DHS populations
(0.07 MB DOC)

Table S2 Questions included from the Domestic Violence module as exposure variables
(0.06 MB DOC)

Table S3 HIV prevalence in each country sample by values of independent variables
(0.18 MB DOC)

Table S4 Prevalence of Intimate Partner Violence in each country sample by values of independent variables
(0.10 MB DOC)

Table S5 Full adjusted regression results for the association between HIV prevalence and each measure of intimate partner violence
(0.30 MB DOC)

Table S6 Unadjusted odds ratios [95% confidence intervals] for HIV prevalence and intimate partner violence by different sample weightings
(0.07 MB DOC)

Table S7 Comparison of main results with and without the Indian sample
(0.10 MB DOC)

Table S8 Main results for unweighted country pooled regressions stratified by marital status
(0.04 MB DOC)

Table S9 Comparison of those refusing and not refusing HIV tests
(0.12 MB DOC)

Table S10 Adjusted odds ratios and 95% confidence intervals for the association between HIV prevalence and measures of intimate partner violence in India
(0.05 MB DOC)
