# Supplementary material for: No Association between HIV and Intimate Partner Violence among Women in 10 Developing Countries
Source: PLoS One. 2010 Dec 8;5(12):e14257. doi: 10.1371/journal.pone.0014257 (PMC2999537; doi:10.1371/journal.pone.0014257)
Supplement: Table S1 — Sampling characteristics of the ten DHS populations (0.07 MB DOC) [file pone.0014257.s001.doc]

**Table S1: Sampling characteristics of the ten DHS populations**

|  | **Pooled** |  | **Dominican Republic** | **Haiti** | **India** | **Kenya** | **Liberia** | **Malawi** | **Mali** | **Rwanda** | **Zambia** | **Zimbabwe** |
| --- | --- | --- | --- | --- | --- | --- | --- | --- | --- | --- | --- | --- |
| **Eligible households** | 225,480 |  | 33,437 | 10,038 | 111,559 | 8,889 | 7,021 | 13,160 | 13,965 | 10,307 | 7,326 | 9,778 |
| Households answering questionnaire | 220,238 |  | 32,431 | 9,998 | 109,041 | 8,561 | 6,824 | 12,998 | 13,664 | 10,272 | 7,164 | 9,285 |
| Response rate | 97.7 |  | 97.0 | 99.6 | 97.7 | 96.3 | 97.2 | 98.8 | 97.8 | 99.7 | 97.8 | 95.0 |
|  |  |  |  |  |  |  |  |  |  |  |  |  |
|  |  |  |  |  |  |  |  |  |  |  |  |  |
| **Eligible women** | 244,004 |  | 29,203 | 10,892 | 131,596 | 8,717 | 7,448 | 15,102 | 12,229 | 11,539 | 7,408 | 9,870 |
| Women answering questionnaire | 231,564 |  | 27,480 | 10,757 | 124,385 | 8,195 | 7,092 | 14,583 | 11,698 | 11,321 | 7,146 | 8,907 |
| Response rate | 94.9 |  | 94.1 | 98.8 | 94.5 | 94.0 | 95.2 | 96.6 | 95.7 | 98.1 | 96.5 | 90.2 |
|  |  |  |  |  |  |  |  |  |  |  |  |  |
|  |  |  |  |  |  |  |  |  |  |  |  |  |
| ***Households selected for HIV test*** |  |  | *All* | *Every* | *See* | *Every* | *All* | *Every* | *Every* | *Every* | *All* | *All* |
|  |  |  |  | *second* | *Footnote^1^* | *second* |  | *third* | *third* | *second* |  |  |
|  |  |  |  |  |  |  |  |  |  |  |  |  |
| **Women eligible for HIV test** | 140,837 |  | 29,203 | 5,368 | 62,182 | 4,303 | 7,448 | 5,157 | 4,071 | 5,827 | 7,408 | 9,870 |
| Women accepting test & result available | 120,092 |  | 25,775 | 5,230 | 52,853 | 3,273 | 6,482 | 4,743 | 2,864 | 5,663 | 5,715 | 7,494 |
| Response rate | 85.3 |  | 88.3 | 97.4 | 85.0 | 76.1 | 87.0 | 92.0 | 70.4 | 97.2 | 77.1 | 75.9 |
|  |  |  |  |  |  |  |  |  |  |  |  |  |
|  |  |  |  |  |  |  |  |  |  |  |  |  |
| ***Households selected for DV module*** |  |  | *Every* | *Every* | *All* | *All* | *All* | *All* | *All* | *Every* | *All* | *All* |
|  |  |  | *second* | *second* |  |  |  |  |  | *second* |  |  |
|  |  |  |  |  |  |  |  |  |  |  |  |  |
| **Women eligible for DV module** | 145,042 |  | 10,416 | 3,575 | 84,268 | 5,977 | 4,995 | 10,272 | 9,863 | 4,066 | 5,259 | 6,351 |
| DV respondents | 117,667 |  | 8,492 | 2,672 | 69,399 | 4,301 | 3,868 | 9,059 | 8,283 | 2,528 | 4,211 | 4,854 |
| Response rate | 81.1 |  | 81.5 | 74.7 | 82.4 | 72.0 | 77.4 | 88.2 | 84.0 | 62.2 | 80.1 | 76.4 |
|  |  |  |  |  |  |  |  |  |  |  |  |  |
|  |  |  |  |  |  |  |  |  |  |  |  |  |
| **Women with valid DV and HIV responses** | 60,795 |  | 7,974 | 2,638 | 29,940 | 1,759 | 3,568 | 2,852 | 2,093 | 2,502 | 3,387 | 4,082 |
| Women missing covariate information | 681 |  | 104 | 10 | 157 | 3 | 290 | 48 | 7 | 26 | 19 | 17 |
|  |  |  |  |  |  |  |  |  |  |  |  |  |
|  |  |  |  |  |  |  |  |  |  |  |  |  |
| **Final analytic sample** | 60,114 |  | 7,870 | 2,628 | 29,783 | 1,756 | 3,278 | 2,804 | 2,086 | 2,476 | 3,368 | 4,065 |

^1^ For the HIV test, India sampled all women in Women’s questionnaire households in seven states (six high-, one low-prevalence) and approximately 20% of eligible households elsewhere.
