# Supplementary material for: No Association between HIV and Intimate Partner Violence among Women in 10 Developing Countries
Source: PLoS One. 2010 Dec 8;5(12):e14257. doi: 10.1371/journal.pone.0014257 (PMC2999537; doi:10.1371/journal.pone.0014257)
Supplement: Table S2 — Questions included from the Domestic Violence module as exposure variables (0.06 MB DOC) [file pone.0014257.s002.doc]

**Table S2: Questions included from the Domestic Violence module as exposure variables**

|  | **Dominican Republic** | **Haiti** | **India** | **Kenya** | **Liberia** | **Mali** | **Malawi** | **Rwanda** | **Zambia** | **Zimbabwe** |
| --- | --- | --- | --- | --- | --- | --- | --- | --- | --- | --- |
| (Does/did) your (last) husband/partner ever: |  |  |  |  |  |  |  |  |  |  |
|  |  |  |  |  |  |  |  |  |  |  |
| **Emotional violence** |  |  |  |  |  |  |  |  |  |  |
| say or do something to humiliate you in front of others? | X | X | X | X | X | X | X | X | X | X |
| threaten to hurt or harm you or someone close to you? | X | X | X | X | X | X | X | X | X | X |
|  |  |  |  |  |  |  |  |  |  |  |
| **Physical violence** |  |  |  |  |  |  |  |  |  |  |
| push you, shake you, or throw something at you? | X | X | X | X | X | X | X | X | X | X |
| slap you? | X | X | X | X | X | X | X | X | X | X |
| punch you with his fist or with something that could hurt you? | X | X | X | X | X | X | X | X | X | X |
| kick you, drag you or beat you up? | X | X | X | X | X | X | X | X | X | X |
| try to choke you or burn you on purpose? | X | X | X | X | X | X | X | X | X | X |
| threaten or attack you with a knife, gun, or any other weapon? | X |  | X | X | X | X | X | X | X | X |
|  |  |  |  |  |  |  |  |  |  |  |
| **Sexual violence** |  |  |  |  |  |  |  |  |  |  |
| physically force you to have sexual intercourse with him even when you did not want to? | X | X | X | X | X | X | X | X | X | X |
| force you to perform any sexual acts you did not want to? | X | X | X | X | X | X | X | X | X | X |
|  |  |  |  |  |  |  |  |  |  |  |
|  |  |  |  |  |  |  |  |  |  |  |
| At least four countries did not ask the following questions, which we therefore did not include in our exposure measures: | | | |  |  |  |  |  |  |  |
|  |  |  |  |  |  |  |  |  |  |  |
| insult you or make you feel bad about yourself? | X | X | X |  | X |  |  |  | X | X |
| twist your arm or pull your hair? | X | X | X |  | X |  |  | X | X |  |

Note: Five countries (Kenya, Mali, Malawi, Rwanda and Zimbabwe) asked two questions regarding weapons, one for threat and another for use. We combined the questions into a single yes/no response for this analysis (yes if either question was responded to in the affirmative).

Note: Haiti did not ask any questions relating to weapons; the physical violence variable may therefore be meaningfully different from that elsewhere.
