# Supplementary material for: No Association between HIV and Intimate Partner Violence among Women in 10 Developing Countries
Source: PLoS One. 2010 Dec 8;5(12):e14257. doi: 10.1371/journal.pone.0014257 (PMC2999537; doi:10.1371/journal.pone.0014257)
Supplement: Table S3 — HIV prevalence in each country sample by values of independent variables (0.18 MB DOC) [file pone.0014257.s003.doc]

**Table S3: HIV prevalence in each country sample by values of independent variables**

|  | Dominican Republic | |  | Haiti | |  | India | |  | Kenya | |  | Liberia | |  | Mali | |  | Malawi | |  | Rwanda | |  | Zambia | |  | Zimbabwe | |
| --- | --- | --- | --- | --- | --- | --- | --- | --- | --- | --- | --- | --- | --- | --- | --- | --- | --- | --- | --- | --- | --- | --- | --- | --- | --- | --- | --- | --- | --- |
|  | N | PLHIV |  | N | PLHIV |  | N | PLHIV |  | N | PLHIV |  | N | PLHIV |  | N | PLHIV |  | N | PLHIV |  | N | PLHIV |  | N | PLHIV |  | N | PLHIV |
| ***Complete-case analytic sample*** | 7,870 | 86 (1.1%) |  | 2,628 | 96 (3.7%) |  | 29,783 | 144 (0.5%) |  | 1,756 | 153 (8.7%) |  | 3,278 | 80 (2.4%) |  | 2,804 | 47 (1.7%) |  | 2,086 | 327 (15.7%) |  | 2,476 | 81 (3.3%) |  | 3,368 | 603 (17.9%) |  | 4,065 | 980 (24.1%) |
| **Age** |  |  |  |  |  |  |  |  |  |  |  |  |  |  |  |  |  |  |  |  |  |  |  |  |  |  |  |  |  |
| 15-19 | 583 | 6 (1.0%) |  | 142 | 4 (2.8%) |  | 1,253 | 7 (0.6%) |  | 132 | 10 (7.6%) |  | 168 | 3 (1.8%) |  | 354 | 4 (1.1%) |  | 185 | 12 (6.5%) |  | 26 | 1 (3.8%) |  | 193 | 18 (9.3%) |  | 250 | 27 (10.8%) |
| 20-24 | 1,197 | 9 (0.8%) |  | 423 | 16 (3.8%) |  | 4,642 | 19 (0.4%) |  | 354 | 36 (10.2%) |  | 550 | 11 (2.0%) |  | 607 | 8 (1.3%) |  | 559 | 87 (15.6%) |  | 454 | 14 (3.1%) |  | 696 | 84 (12.1%) |  | 885 | 139 (15.7%) |
| 25-29 | 1,426 | 12 (0.8%) |  | 556 | 28 (5.0%) |  | 6,459 | 33 (0.5%) |  | 392 | 45 (11.5%) |  | 637 | 18 (2.8%) |  | 570 | 8 (1.4%) |  | 467 | 78 (16.7%) |  | 585 | 20 (3.4%) |  | 846 | 165 (19.5%) |  | 895 | 234 (26.1%) |
| 30-34 | 1,473 | 25 (1.7%) |  | 453 | 19 (4.2%) |  | 6,235 | 43 (0.7%) |  | 367 | 31 (8.4%) |  | 640 | 14 (2.2%) |  | 440 | 11 (2.5%) |  | 339 | 62 (18.3%) |  | 587 | 23 (3.9%) |  | 660 | 148 (22.4%) |  | 781 | 250 (32.0%) |
| 35-39 | 1,301 | 16 (1.2%) |  | 414 | 10 (2.4%) |  | 5,005 | 19 (0.4%) |  | 235 | 15 (6.4%) |  | 570 | 19 (3.3%) |  | 357 | 7 (2.0%) |  | 223 | 41 (18.4%) |  | 340 | 12 (3.5%) |  | 421 | 99 (23.5%) |  | 514 | 163 (31.7%) |
| 40-44 | 1,022 | 10 (1.0%) |  | 324 | 12 (3.7%) |  | 3,638 | 14 (0.4%) |  | 167 | 13 (7.8%) |  | 386 | 8 (2.1%) |  | 275 | 4 (1.5%) |  | 177 | 29 (16.4%) |  | 274 | 7 (2.6%) |  | 298 | 54 (18.1%) |  | 411 | 105 (25.5%) |
| 45-49 | 868 | 8 (0.9%) |  | 316 | 7 (2.2%) |  | 2,551 | 9 (0.4%) |  | 109 | 3 (2.8%) |  | 327 | 7 (2.1%) |  | 201 | 5 (2.5%) |  | 136 | 18 (13.2%) |  | 210 | 4 (1.9%) |  | 254 | 35 (13.8%) |  | 329 | 62 (18.8%) |
| **Marital status** |  |  |  |  |  |  |  |  |  |  |  |  |  |  |  |  |  |  |  |  |  |  |  |  |  |  |  |  |  |
| Currently | 6,232 | 62 (1.0%) |  | 2,298 | 79 (3.4%) |  | 28,008 | 95 (0.3%) |  | 1,618 | 123 (7.6%) |  | 2,937 | 66 (2.2%) |  | 2,709 | 43 (1.6%) |  | 1,875 | 269 (14.3%) |  | 2,268 | 65 (2.9%) |  | 2,870 | 431 (15.0%) |  | 3,388 | 669 (19.7%) |
| Formerly | 1,638 | 24 (1.5%) |  | 330 | 17 (5.2%) |  | 1,775 | 49 (2.8%) |  | 138 | 30 (21.7%) |  | 341 | 14 (4.1%) |  | 95 | 4 (4.2%) |  | 211 | 58 (27.5%) |  | 208 | 16 (7.7%) |  | 498 | 172 (34.5%) |  | 677 | 311 (45.9%) |
| **Urbanity** |  |  |  |  |  |  |  |  |  |  |  |  |  |  |  |  |  |  |  |  |  |  |  |  |  |  |  |  |  |
| Urban | 4,523 | 45 (1.0%) |  | 1,103 | 48 (4.4%) |  | 13,953 | 75 (0.5%) |  | 462 | 62 (13.4%) |  | 1,092 | 46 (4.2%) |  | 881 | 16 (1.8%) |  | 220 | 53 (24.1%) |  | 400 | 35 (8.8%) |  | 1,196 | 345 (28.8%) |  | 1,109 | 287 (25.9%) |
| Rural | 3,347 | 41 (1.2%) |  | 1,525 | 48 (3.1%) |  | 15,830 | 69 (0.4%) |  | 1,294 | 91 (7.0%) |  | 2,186 | 34 (1.6%) |  | 1,923 | 31 (1.6%) |  | 1,866 | 274 (14.7%) |  | 2,076 | 46 (2.2%) |  | 2,172 | 258 (11.9%) |  | 2,956 | 693 (23.4%) |
| **Wealth quintiles** |  |  |  |  |  |  |  |  |  |  |  |  |  |  |  |  |  |  |  |  |  |  |  |  |  |  |  |  |  |
| Poorest | 2,256 | 44 (2.0%) |  | 599 | 17 (2.8%) |  | 3,646 | 19 (0.5%) |  | 361 | 15 (4.2%) |  | 835 | 10 (1.2%) |  | 485 | 11 (2.3%) |  | 368 | 45 (12.2%) |  | 521 | 15 (2.9%) |  | 649 | 53 (8.2%) |  | 924 | 199 (21.5%) |
| 2nd poorest | 1,889 | 20 (1.1%) |  | 539 | 11 (2.0%) |  | 4,885 | 22 (0.5%) |  | 335 | 25 (7.5%) |  | 760 | 15 (2.0%) |  | 553 | 9 (1.6%) |  | 470 | 50 (10.6%) |  | 522 | 12 (2.3%) |  | 701 | 77 (11.0%) |  | 866 | 194 (22.4%) |
| Middle | 1,550 | 11 (0.7%) |  | 538 | 21 (3.9%) |  | 6,314 | 30 (0.5%) |  | 317 | 24 (7.6%) |  | 660 | 13 (2.0%) |  | 601 | 5 (0.8%) |  | 518 | 85 (16.4%) |  | 491 | 15 (3.1%) |  | 760 | 116 (15.3%) |  | 755 | 194 (25.7%) |
| 2nd richest | 1,301 | 3 (0.2%) |  | 584 | 36 (6.2%) |  | 7,225 | 50 (0.7%) |  | 343 | 33 (9.6%) |  | 607 | 26 (4.3%) |  | 643 | 12 (1.9%) |  | 451 | 83 (18.4%) |  | 545 | 15 (2.8%) |  | 764 | 201 (26.3%) |  | 908 | 256 (28.2%) |
| Richest | 874 | 8 (0.9%) |  | 368 | 11 (3.0%) |  | 7,713 | 23 (0.3%) |  | 400 | 56 (14.0%) |  | 416 | 16 (3.8%) |  | 522 | 10 (1.9%) |  | 279 | 64 (22.9%) |  | 397 | 24 (6.0%) |  | 494 | 156 (31.6%) |  | 612 | 137 (22.4%) |
| **Education** |  |  |  |  |  |  |  |  |  |  |  |  |  |  |  |  |  |  |  |  |  |  |  |  |  |  |  |  |  |
| None | 481 | 17 (3.5%) |  | 939 | 25 (2.7%) |  | 11,626 | 67 (0.6%) |  | 342 | 10 (2.9%) |  | 1,754 | 28 (1.6%) |  | 2,312 | 40 (1.7%) |  | 565 | 81 (14.3%) |  | 711 | 19 (2.7%) |  | 459 | 48 (10.5%) |  | 212 | 43 (20.3%) |
| Primary | 3,697 | 53 (1.4%) |  | 1,022 | 44 (4.3%) |  | 4,661 | 25 (0.5%) |  | 966 | 97 (10.0%) |  | 1,024 | 33 (3.2%) |  | 321 | 5 (1.6%) |  | 1,333 | 206 (15.5%) |  | 1,562 | 55 (3.5%) |  | 2,013 | 327 (16.2%) |  | 1,593 | 382 (24.0%) |
| Secondary & above | 3,692 | 16 (0.4%) |  | 667 | 27 (4.0%) |  | 13,496 | 52 (0.4%) |  | 448 | 46 (10.3%) |  | 500 | 19 (3.8%) |  | 171 | 2 (1.2%) |  | 188 | 40 (21.3%) |  | 203 | 7 (3.4%) |  | 896 | 228 (25.4%) |  | 2,260 | 555 (24.6%) |
| **Occupation** |  |  |  |  |  |  |  |  |  |  |  |  |  |  |  |  |  |  |  |  |  |  |  |  |  |  |  |  |  |
| Not employed | 4,019 | 49 (1.2%) |  | 810 | 26 (3.2%) |  | 16,430 | 67 (0.4%) |  | 592 | 52 (8.8%) |  | 810 | 23 (2.8%) |  | 1,228 | 18 (1.5%) |  | 776 | 127 (16.4%) |  | 544 | 17 (3.1%) |  | 1,328 | 255 (19.2%) |  | 2,195 | 512 (23.3%) |
| Manual | 759 | 4 (0.5%) |  | 79 | 1 (1.3%) |  | 3,040 | 16 (0.5%) |  | 61 | 9 (14.8%) |  | 30 | 1 (3.3%) |  | 191 | 1 (0.5%) |  | 49 | 13 (26.5%) |  | 45 | 4 (8.9%) |  | 135 | 28 (20.7%) |  | 219 | 52 (23.7%) |
| Agricultural |  |  |  | 338 | 6 (1.8%) |  | 6,831 | 26 (0.4%) |  | 640 | 37 (5.8%) |  | 1,544 | 15 (1.0%) |  | 789 | 12 (1.5%) |  | 992 | 124 (12.5%) |  | 1,747 | 46 (2.6%) |  | 1,121 | 115 (10.3%) |  | 830 | 186 (22.4%) |
| Non-manual, non-agricultural ^†^ | 3,092 | 33 (1.1%) |  | 1,401 | 63 (4.5%) |  | 3,482 | 35 (1.0%) |  | 463 | 55 (11.9%) |  | 894 | 41 (4.6%) |  | 596 | 16 (2.7%) |  | 269 | 63 (23.4%) |  | 140 | 14 (10.0%) |  | 784 | 205 (26.1%) |  | 821 | 230 (28.0%) |
| **Religion** |  |  |  |  |  |  |  |  |  |  |  |  |  |  |  |  |  |  |  |  |  |  |  |  |  |  |  |  |  |
| Christian |  |  |  | 2,452 | 91 (3.7%) |  | 1,577 | 8 (0.5%) |  | 1,477 | 143 (9.7%) |  | 2,773 | 69 (2.5%) |  | 100 | 3 (3.0%) |  | 1,712 | 263 (15.4%) |  | 2,395 | 73 (3.0%) |  | 3,304 | 594 (18.0%) |  | 3,507 | 845 (24.1%) |
| Muslim |  |  |  |  |  |  | 3,613 | 7 (0.2%) |  | 235 | 8 (3.4%) |  | 396 | 10 (2.5%) |  | 2,570 | 40 (1.6%) |  | 354 | 63 (17.8%) |  | 44 | 6 (13.6%) |  | 15 | 1 (6.7%) |  | 26 | 6 (23.1%) |
| Hindu |  |  |  |  |  |  | 23,321 | 122 (0.5%) |  |  |  |  |  |  |  |  |  |  |  |  |  |  |  |  |  |  |  |  |  |
| Other/none |  |  |  | 176 | 5 (2.8%) |  | 1,272 | 7 (0.6%) |  | 44 | 2 (4.5%) |  | 109 | 1 (0.9%) |  | 134 | 4 (3.0%) |  | 20 | 1 (5.0%) |  | 37 | 2 (5.4%) |  | 49 | 8 (16.3%) |  | 532 | 129 (24.2%) |
| **Lifetime # of partners** |  |  |  |  |  |  |  |  |  |  |  |  |  |  |  |  |  |  |  |  |  |  |  |  |  |  |  |  |  |
| Zero or one | 3,764 | 10 (0.3%) |  | 1,115 | 14 (1.3%) |  | 29,292 | 131 (0.4%) |  |  |  |  | 606 | 8 (1.3%) |  | 2,056 | 21 (1.0%) |  |  |  |  | 1,806 | 35 (1.9%) |  | 1,431 | 129 (9.0%) |  | 2,660 | 466 (17.5%) |
| Two or more | 4,106 | 76 (1.9%) |  | 1,513 | 82 (5.4%) |  | 491 | 13 (2.6%) |  |  |  |  | 2,672 | 72 (2.7%) |  | 748 | 26 (3.5%) |  |  |  |  | 670 | 46 (6.9%) |  | 1,937 | 474 (24.5%) |  | 1,405 | 514 (36.6%) |
|  |  |  |  |  |  |  |  |  |  |  |  |  |  |  |  |  |  |  |  |  |  |  |  |  |  |  |  |  |  |
| **Intimate partner violence ^*^** |  |  |  |  |  |  |  |  |  |  |  |  |  |  |  |  |  |  |  |  |  |  |  |  |  |  |  |  |  |
| No physical nor sexual violence | 6,562 | 67 (1%) |  | 2,171 | 86 (4.0%) |  | 19,285 | 75 (0.4%) |  | 999 | 88 (8.8%) |  | 2,010 | 49 (2.4%) |  | 2,322 | 37 (1.6%) |  | 1498 | 229 (15.3%) |  | 1,607 | 49 (3.0%) |  | 2,632 | 631 (24.0%) |  | 1,732 | 295 (17.0%) |
|  |  |  |  |  |  |  |  |  |  |  |  |  |  |  |  |  |  |  |  |  |  |  |  |  |  |  |  |  |  |
| Any physical or sexual violence | 1,308 | 19 (1.5%) |  | 457 | 10 (2.2%) |  | 10,498 | 69 (0.7%) |  | 757 | 65 (8.6%) |  | 1,268 | 31 (2.4%) |  | 482 | 10 (2.1%) |  | 588 | 98 (16.7%) |  | 869 | 32 (3.7%) |  | 1,433 | 349 (24.4%) |  | 1,636 | 308 (18.8%) |
| Any physical violence | 1,238 | 19 (1.5%) |  | 312 | 8 (2.6%) |  | 10,170 | 66 (0.6%) |  | 700 | 60 (8.6%) |  | 1,177 | 29 (2.5%) |  | 453 | 9 (2.0%) |  | 451 | 77 (17.1%) |  | 770 | 28 (3.6%) |  | 1,514 | 290 (19.2%) |  | 1,226 | 309 (25.2%) |
| Any sexual violence | 435 | 6 (1.4%) |  | 278 | 5 (1.8%) |  | 2,057 | 18 (0.9%) |  | 252 | 17 (6.7%) |  | 302 | 6 (2.0%) |  | 99 | 3 (3.0%) |  | 291 | 46 (15.8%) |  | 344 | 16 (4.7%) |  | 573 | 122 (21.3%) |  | 566 | 128 (22.6%) |
| Physical and sexual violence | 365 | 6 (1.6%) |  | 133 | 3 (2.3%) |  | 1,729 | 15 (0.9%) |  | 195 | 12 (6.2%) |  | 211 | 4 (1.9%) |  | 70 | 2 (2.9%) |  | 154 | 25 (16.2%) |  | 245 | 12 (4.9%) |  | 451 | 104 (23.1%) |  | 359 | 88 (24.5%) |

Notes. PLHIV = Persons living with HIV (percentages for PLHIV are of the relevant row N). The Dominican Republic did not ask about religion and neither Kenya nor Malawi asked about lifetime number of partners.

^†^ In the Dominican Republic only 113 women reported working in agriculture and none of them were PLHIV; we added them to the non-manual category so as to keep them in the analysis.
^*^ Each item of ‘Intimate partner violence’ is a binary measure of those women reporting any issues in the category.
