# Supplementary material for: No Association between HIV and Intimate Partner Violence among Women in 10 Developing Countries
Source: PLoS One. 2010 Dec 8;5(12):e14257. doi: 10.1371/journal.pone.0014257 (PMC2999537; doi:10.1371/journal.pone.0014257)
Supplement: Table S4 — Prevalence of Intimate Partner Violence in each country sample by values of independent variables (0.10 MB DOC) [file pone.0014257.s004.doc]

**Table S4: Prevalence of Intimate Partner Violence in each country sample by values of independent variables**

|  | **Dominican Republic** | |  | **Haiti** | |  | **India** | |  | **Kenya** | |  | **Liberia** | |
| --- | --- | --- | --- | --- | --- | --- | --- | --- | --- | --- | --- | --- | --- | --- |
|  | **N** | **Physical  or sexual  violence** |  | **N** | **Physical  or sexual  violence** |  | **N** | **Physical  or sexual  violence** |  | **N** | **Physical  or sexual  violence** |  | **N** | **Physical  or sexual  violence** |
|  |  |  |  |  |  |  |  |  |  |  |  |  |  |  |
| **Analytic sample** | 7,870 | 1,308 (16.6%) |  | 2,628 | 457 (17.4%) |  | 29,783 | 10,498 (35.2%) |  | 1,756 | 757 (43.1%) |  | 3,278 | 1,268 (38.7%) |
| **Age** |  |  |  |  |  |  |  |  |  |  |  |  |  |  |
| 15-19 | 583 | 95 (16.3%) |  | 142 | 28 (19.7%) |  | 1,253 | 337 (26.9%) |  | 132 | 37 (28.0%) |  | 168 | 72 (42.9%) |
| 20-24 | 1,197 | 250 (20.9%) |  | 423 | 75 (17.7%) |  | 4,642 | 1,549 (33.4%) |  | 354 | 142 (40.1%) |  | 550 | 259 (47.1%) |
| 25-29 | 1,426 | 252 (17.7%) |  | 556 | 102 (18.3%) |  | 6,459 | 2,298 (35.6%) |  | 392 | 164 (41.8%) |  | 637 | 294 (46.2%) |
| 30-34 | 1,473 | 244 (16.6%) |  | 453 | 88 (19.4%) |  | 6,235 | 2,278 (36.5%) |  | 367 | 160 (43.6%) |  | 640 | 244 (38.1%) |
| 35-39 | 1,301 | 199 (15.3%) |  | 414 | 63 (15.2%) |  | 5,005 | 1,874 (37.4%) |  | 235 | 122 (51.9%) |  | 570 | 189 (33.2%) |
| 40-44 | 1,022 | 144 (14.1%) |  | 324 | 57 (17.6%) |  | 3,638 | 1,282 (35.2%) |  | 167 | 85 (50.9%) |  | 386 | 107 (27.7%) |
| 45-49 | 868 | 124 (14.3%) |  | 316 | 44 (13.9%) |  | 2,551 | 880 (34.5%) |  | 109 | 47 (43.1%) |  | 327 | 103 (31.5%) |
| **Marital status** |  |  |  |  |  |  |  |  |  |  |  |  |  |  |
| Currently | 6,232 | 916 (14.7%) |  | 2,298 | 386 (16.8%) |  | 28,008 | 9,670 (34.5%) |  | 1,618 | 674 (41.7%) |  | 2,937 | 1,092 (37.2%) |
| Formerly | 1,638 | 392 (23.9%) |  | 330 | 71 (21.5%) |  | 1,775 | 828 (46.6%) |  | 138 | 83 (60.1%) |  | 341 | 176 (51.6%) |
| **Urbanity** |  |  |  |  |  |  |  |  |  |  |  |  |  |  |
| Urban | 4,523 | 761 (16.8%) |  | 1,103 | 214 (19.4%) |  | 13,953 | 4,465 (32.0%) |  | 462 | 180 (39.0%) |  | 1,092 | 480 (44.0%) |
| Rural | 3,347 | 547 (16.3%) |  | 1,525 | 243 (15.9%) |  | 15,830 | 6,033 (38.1%) |  | 1,294 | 577 (44.6%) |  | 2,186 | 788 (36.0%) |
| **Wealth quintiles** |  |  |  |  |  |  |  |  |  |  |  |  |  |  |
| Poorest | 2,256 | 415 (18.4%) |  | 599 | 95 (15.9%) |  | 3,646 | 1,753 (48.1%) |  | 361 | 154 (42.7%) |  | 835 | 303 (36.3%) |
| 2nd poorest | 1,889 | 368 (19.5%) |  | 539 | 90 (16.7%) |  | 4,885 | 2,211 (45.3%) |  | 335 | 159 (47.5%) |  | 760 | 300 (39.5%) |
| Middle | 1,550 | 280 (18.1%) |  | 538 | 102 (19.0%) |  | 6,314 | 2,540 (40.2%) |  | 317 | 148 (46.7%) |  | 660 | 249 (37.7%) |
| 2nd richest | 1,301 | 156 (12.0%) |  | 584 | 111 (19.0%) |  | 7,225 | 2,550 (35.3%) |  | 343 | 150 (43.7%) |  | 607 | 257 (42.3%) |
| Richest | 874 | 89 (10.2%) |  | 368 | 59 (16.0%) |  | 7,713 | 1,444 (18.7%) |  | 400 | 146 (36.5%) |  | 416 | 159 (38.2%) |
| **Education** |  |  |  |  |  |  |  |  |  |  |  |  |  |  |
| None | 481 | 79 (16.4%) |  | 939 | 161 (17.1%) |  | 11,626 | 5,192 (44.7%) |  | 342 | 133 (38.9%) |  | 1,754 | 645 (36.8%) |
| Primary | 3,697 | 694 (18.8%) |  | 1,022 | 198 (19.4%) |  | 4,661 | 1,904 (40.8%) |  | 966 | 463 (47.9%) |  | 1,024 | 422 (41.2%) |
| Secondary & above | 3,692 | 535 (14.5%) |  | 667 | 98 (14.7%) |  | 13,496 | 3,402 (25.2%) |  | 448 | 161 (35.9%) |  | 500 | 201 (40.2%) |
| **Occupation** |  |  |  |  |  |  |  |  |  |  |  |  |  |  |
| Not employed | 4,019 | 594 (14.8%) |  | 810 | 152 (18.8%) |  | 16,430 | 4,961 (30.2%) |  | 592 | 214 (36.1%) |  | 810 | 343 (42.3%) |
| Agricultural |  |  |  | 338 | 51 (15.1%) |  | 6,831 | 3,036 (44.4%) |  | 640 | 322 (50.3%) |  | 1,544 | 535 (34.7%) |
| Manual | 759 | 142 (18.7%) |  | 79 | 6 (7.6%) |  | 3,040 | 1,282 (42.2%) |  | 61 | 19 (31.1%) |  | 30 | 15 (50.0%) |
| Non-manual, non-agricultural ^†^ | 3,092 | 548 (17.7%) |  | 1,401 | 248 (17.7%) |  | 3,482 | 1,219 (35.0%) |  | 463 | 202 (43.6%) |  | 894 | 375 (41.9%) |
| **Religion** |  |  |  |  |  |  |  |  |  |  |  |  |  |  |
| Christian |  |  |  | 2,452 | 402 (16.4%) |  | 1,577 | 510 (32.3%) |  | 1,477 | 670 (45.4%) |  | 2,773 | 1,090 (39.3%) |
| Muslim |  |  |  |  |  |  | 3,613 | 1,348 (37.3%) |  | 235 | 76 (32.3%) |  | 396 | 129 (32.6%) |
| Hindu |  |  |  |  |  |  | 23,321 | 8,178 (35.1%) |  |  |  |  |  |  |
| Other/none |  |  |  | 176 | 55 (31.3%) |  | 1,272 | 462 (36.3%) |  | 44 | 11 (25.0%) |  | 109 | 49 (45.0%) |
| **Lifetime # of partners** |  |  |  |  |  |  |  |  |  |  |  |  |  |  |
| Zero or one | 3,764 | 469 (12.5%) |  | 1,115 | 169 (15.2%) |  | 29,292 | 10,243 (35.0%) |  |  |  |  | 606 | 215 (35.5%) |
| Two or more | 4,106 | 839 (20.4%) |  | 1,513 | 288 (19.0%) |  | 491 | 255 (51.9%) |  |  |  |  | 2,672 | 1,053 (39.4%) |

^†^ In the Dominican Republic only 113 women reported working in agriculture and none of them were PLHIV; we added them to the non-manual category so as to keep them in the analysis.
