# Supplementary material for: No Association between HIV and Intimate Partner Violence among Women in 10 Developing Countries
Source: PLoS One. 2010 Dec 8;5(12):e14257. doi: 10.1371/journal.pone.0014257 (PMC2999537; doi:10.1371/journal.pone.0014257)
Supplement: Table S5 — Full adjusted regression results for the association between HIV prevalence and each measure of intimate partner violence (0.30 MB DOC) [file pone.0014257.s005.doc]

**Table S5: Full adjusted regression results for the association between HIV prevalence and each measure of intimate partner violence**

|  | Dominican Republic | Haiti | India | Kenya | Liberia | Malawi | Mali | Rwanda | Zambia | Zimbabwe | Pooled ^*^ |
| --- | --- | --- | --- | --- | --- | --- | --- | --- | --- | --- | --- |
| **Any physical violence** | 1.19 | 0.52 | 1.30 | 0.89 | 0.88 | 0.99 | 1.08 | 1.03 | 0.95 | 1.02 | 1.06 |
| **vs. no physical violence** | [0.71 - 1.99] | [0.24 - 1.10] | [0.93 - 1.82] | [0.63 - 1.25] | [0.55 - 1.39] | [0.46 - 2.13] | [0.80 - 1.45] | [0.64 - 1.66] | [0.79 - 1.15] | [0.86 - 1.21] | [0.96 - 1.16] |
| Age 15-19 |  |  |  |  |  |  |  |  |  |  |  |
|  |  |  |  |  |  |  |  |  |  |  |  |
| Age 20-24 | 0.69 | 1.34 | 0.65 | 1.32 | 1.06 | 1.16 | 2.53 | 0.92 | 1.19 | 1.36 | 1.37 |
|  | [0.24 - 1.97] | [0.41 - 4.33] | [0.27 - 1.56] | [0.63 - 2.79] | [0.28 - 3.99] | [0.25 - 5.37] | [1.39 - 4.60] | [0.09 - 9.70] | [0.66 - 2.14] | [0.90 - 2.04] | [1.07 - 1.74] |
| Age 25-29 | 0.79 | 1.61 | 0.73 | 1.62 | 1.51 | 1.24 | 2.66 | 0.77 | 1.92 | 2.60 | 2.06 |
|  | [0.29 - 2.13] | [0.54 - 4.80] | [0.33 - 1.61] | [0.80 - 3.28] | [0.40 - 5.77] | [0.28 - 5.49] | [1.44 - 4.92] | [0.08 - 7.28] | [1.14 - 3.23] | [1.73 - 3.93] | [1.63 - 2.60] |
| Age 30-34 | 1.53 | 1.34 | 0.85 | 1.12 | 1.13 | 1.93 | 2.93 | 0.85 | 2.39 | 3.31 | 2.39 |
|  | [0.62 - 3.75] | [0.42 - 4.26] | [0.38 - 1.87] | [0.54 - 2.30] | [0.32 - 4.02] | [0.44 - 8.44] | [1.52 - 5.65] | [0.09 - 7.78] | [1.38 - 4.13] | [2.14 - 5.11] | [1.87 - 3.04] |
| Age 35-39 | 1.10 | 0.68 | 0.36 | 0.85 | 1.81 | 1.44 | 3.15 | 0.79 | 2.62 | 2.88 | 2.12 |
|  | [0.44 - 2.75] | [0.20 - 2.31] | [0.14 - 0.90] | [0.33 - 2.20] | [0.51 - 6.36] | [0.39 - 5.23] | [1.70 - 5.85] | [0.08 - 8.31] | [1.55 - 4.41] | [1.91 - 4.33] | [1.68 - 2.68] |
| Age 40-44 | 0.80 | 1.10 | 0.32 | 1.27 | 1.34 | 0.99 | 2.64 | 0.61 | 1.60 | 2.01 | 1.64 |
|  | [0.28 - 2.28] | [0.33 - 3.68] | [0.13 - 0.82] | [0.49 - 3.27] | [0.32 - 5.69] | [0.17 - 5.61] | [1.23 - 5.64] | [0.07 - 5.03] | [0.86 - 2.98] | [1.25 - 3.23] | [1.25 - 2.14] |
| Age 45-49 | 0.62 | 0.71 | 0.24 | 0.43 | 1.41 | 1.73 | 1.93 | 0.58 | 1.25 | 1.28 | 1.18 |
|  | [0.21 - 1.87] | [0.19 - 2.68] | [0.10 - 0.60] | [0.12 - 1.55] | [0.33 - 6.08] | [0.33 - 9.02] | [0.89 - 4.18] | [0.05 - 7.27] | [0.69 - 2.28] | [0.79 - 2.07] | [0.90 - 1.56] |
| Previously vs. currently married | 1.25 | 1.44 | 8.82 | 3.40 | 1.53 | 2.34 | 2.64 | 1.87 | 2.46 | 3.08 | 2.98 |
|  | [0.75 - 2.10] | [0.83 - 2.49] | [5.94 - 13.10] | [2.07 - 5.59] | [0.82 - 2.87] | [0.77 - 7.07] | [1.82 - 3.82] | [1.04 - 3.37] | [1.96 - 3.09] | [2.54 - 3.73] | [2.68 - 3.31] |
| Rural vs. urban residence | 0.87 | 1.26 | 0.86 | 0.69 | 0.67 | 1.19 | 0.71 | 0.33 | 0.69 | 0.81 | 0.76 |
|  | [0.53 - 1.44] | [0.79 - 2.02] | [0.56 - 1.32] | [0.43 - 1.11] | [0.37 - 1.22] | [0.42 - 3.34] | [0.48 - 1.05] | [0.19 - 0.58] | [0.48 - 0.99] | [0.61 - 1.07] | [0.66 - 0.88] |
| Poorest quintile |  |  |  |  |  |  |  |  |  |  |  |
|  |  |  |  |  |  |  |  |  |  |  |  |
| 2^nd^ poorest quintile | 0.67 | 0.71 | 0.89 | 1.36 | 1.26 | 0.77 | 1.03 | 0.74 | 1.26 | 1.08 | 0.99 |
|  | [0.38 - 1.20] | [0.31 - 1.63] | [0.46 - 1.73] | [0.65 - 2.86] | [0.51 - 3.07] | [0.34 - 1.71] | [0.64 - 1.67] | [0.34 - 1.61] | [0.89 - 1.78] | [0.86 - 1.37] | [0.86 - 1.15] |
| Middle quintile | 0.51 | 1.20 | 0.93 | 1.37 | 0.88 | 0.39 | 1.74 | 0.95 | 1.59 | 1.28 | 1.16 |
|  | [0.24 - 1.06] | [0.53 - 2.69] | [0.51 - 1.68] | [0.74 - 2.52] | [0.36 - 2.14] | [0.14 - 1.12] | [1.14 - 2.66] | [0.41 - 2.17] | [1.00 - 2.53] | [1.02 - 1.61] | [1.00 - 1.34] |
| 2^nd^ richest quintile | 0.19 | 1.91 | 1.55 | 1.43 | 1.19 | 0.84 | 1.95 | 0.84 | 2.58 | 1.25 | 1.37 |
|  | [0.05 - 0.66] | [0.88 - 4.13] | [0.84 - 2.88] | [0.70 - 2.94] | [0.47 - 2.99] | [0.32 - 2.25] | [1.24 - 3.09] | [0.33 - 2.10] | [1.52 - 4.35] | [0.95 - 1.66] | [1.16 - 1.61] |
| Richest quintile | 1.02 | 0.93 | 0.82 | 1.54 | 0.90 | 0.99 | 2.03 | 0.91 | 2.92 | 0.78 | 1.16 |
|  | [0.42 - 2.47] | [0.35 - 2.48] | [0.37 - 1.80] | [0.70 - 3.42] | [0.33 - 2.46] | [0.25 - 4.01] | [1.23 - 3.36] | [0.35 - 2.35] | [1.64 - 5.18] | [0.51 - 1.18] | [0.94 - 1.42] |
| No education |  |  |  |  |  |  |  |  |  |  |  |
|  |  |  |  |  |  |  |  |  |  |  |  |
| Any primary | 0.54 | 1.34 | 0.78 | 2.40 | 1.92 | 0.74 | 1.04 | 1.28 | 1.27 | 1.23 | 1.19 |
|  | [0.30 - 0.98] | [0.79 - 2.28] | [0.50 - 1.22] | [0.99 - 5.83] | [1.12 - 3.29] | [0.30 - 1.86] | [0.78 - 1.40] | [0.69 - 2.37] | [0.89 - 1.82] | [0.86 - 1.75] | [1.04 - 1.37] |
| More than primary | 0.22 | 1.15 | 0.63 | 1.82 | 1.37 | 0.50 | 1.05 | 0.59 | 1.26 | 1.29 | 1.05 |
|  | [0.09 - 0.50] | [0.57 - 2.35] | [0.40 - 0.99] | [0.71 - 4.65] | [0.60 - 3.11] | [0.11 - 2.34] | [0.61 - 1.81] | [0.18 - 1.98] | [0.82 - 1.95] | [0.89 - 1.86] | [0.89 - 1.24] |
| No employment |  |  |  |  |  |  |  |  |  |  |  |
|  |  |  |  |  |  |  |  |  |  |  |  |
| Non-manual, non-agricultural | 0.93 | 1.37 | 1.43 | 0.92 | 1.41 | 1.47 | 1.27 | 2.47 | 0.87 | 0.90 | 1.12 |
|  | [0.59 - 1.47] | [0.85 - 2.20] | [0.92 - 2.22] | [0.59 - 1.43] | [0.79 - 2.50] | [0.74 - 2.93] | [0.84 - 1.91] | [0.86 - 7.13] | [0.66 - 1.14] | [0.72 - 1.11] | [0.99 - 1.26] |
| Manual | 0.40 | 0.37 | 0.79 | 1.25 | 1.15 | 0.31 | 1.65 | 1.92 | 0.80 | 0.88 | 0.90 |
|  | [0.14 - 1.15] | [0.05 - 2.87] | [0.46 - 1.33] | [0.55 - 2.84] | [0.15 - 9.04] | [0.03 - 2.71] | [0.80 - 3.42] | [0.50 - 7.40] | [0.44 - 1.48] | [0.62 - 1.25] | [0.73 - 1.12] |
| Agricultural |  | 0.78 | 0.66 | 0.56 | 0.42 | 0.87 | 0.79 | 1.05 | 0.66 | 0.91 | 0.72 |
|  |  | [0.30 - 1.99] | [0.40 - 1.08] | [0.36 - 0.88] | [0.21 - 0.86] | [0.43 - 1.78] | [0.60 - 1.04] | [0.55 - 2.02] | [0.52 - 0.85] | [0.73 - 1.14] | [0.64 - 0.81] |
| Christian |  |  |  |  |  |  |  |  |  |  |  |
|  |  |  |  |  |  |  |  |  |  |  |  |
| Hindu |  |  | 1.19 |  |  |  |  |  |  |  |  |
|  |  |  | [0.52 - 2.69] |  |  |  |  |  |  |  |  |
| Muslim |  |  | 0.36 | 0.37 | 1.05 | 0.73 | 1.24 | 2.42 |  | 0.96 |  |
|  |  |  | [0.12 - 1.10] | [0.15 - 0.91] | [0.55 - 2.00] | [0.23 - 2.30] | [0.92 - 1.68] | [0.86 - 6.78] |  | [0.35 - 2.64] |  |
| Other/no religion |  | 0.74 | 1.20 | 0.55 | 0.50 | 1.19 | 0.29 | 1.58 |  | 1.04 |  |
|  |  | [0.29 - 1.94] | [0.40 - 3.58] | [0.13 - 2.35] | [0.06 - 3.95] | [0.29 - 4.89] | [0.04 - 2.23] | [0.37 - 6.75] |  | [0.81 - 1.35] |  |
| >1 vs. 0-1 lifetime sexual partners | 5.77 | 4.59 | 4.18 |  | 1.59 | 3.38 |  | 3.00 | 2.89 | 2.22 |  |
|  | [2.93 - 11.37] | [2.58 - 8.19] | [2.26 - 7.72] |  | [0.78 - 3.22] | [1.72 - 6.64] |  | [1.72 - 5.23] | [2.24 - 3.71] | [1.88 - 2.63] |  |

^*^ Country-level fixed effects are included for the pooled regression, but values are not shown in this table.

**Table S5: Full adjusted regression results for the association between HIV prevalence and each measure of intimate partner violence**

|  | Dominican Republic | Haiti | India | Kenya | Liberia | Malawi | Mali | Rwanda | Zambia | Zimbabwe | Pooled ^*^ |
| --- | --- | --- | --- | --- | --- | --- | --- | --- | --- | --- | --- |
| **Any sexual violence** | 0.96 | 0.39 | 1.27 | 0.61 | 0.74 | 1.64 | 1.00 | 0.99 | 0.99 | 0.90 | 0.98 |
| **vs. no sexual violence** | [0.40 - 2.31] | [0.15 - 1.00] | [0.75 - 2.13] | [0.35 - 1.05] | [0.32 - 1.73] | [0.40 - 6.68] | [0.70 - 1.43] | [0.55 - 1.77] | [0.78 - 1.26] | [0.70 - 1.16] | [0.87 - 1.11] |
| Age 15-19 |  |  |  |  |  |  |  |  |  |  |  |
|  |  |  |  |  |  |  |  |  |  |  |  |
| Age 20-24 | 0.70 | 1.30 | 0.67 | 1.34 | 1.07 | 1.17 | 2.54 | 0.93 | 1.18 | 1.35 | 1.37 |
|  | [0.25 - 2.00] | [0.40 - 4.18] | [0.28 - 1.60] | [0.63 - 2.83] | [0.29 - 4.01] | [0.25 - 5.50] | [1.39 - 4.62] | [0.09 - 9.79] | [0.66 - 2.12] | [0.90 - 2.03] | [1.08 - 1.75] |
| Age 25-29 | 0.79 | 1.56 | 0.75 | 1.65 | 1.53 | 1.27 | 2.67 | 0.77 | 1.90 | 2.59 | 2.07 |
|  | [0.29 - 2.14] | [0.52 - 4.65] | [0.34 - 1.67] | [0.81 - 3.37] | [0.40 - 5.84] | [0.29 - 5.62] | [1.44 - 4.93] | [0.08 - 7.33] | [1.13 - 3.19] | [1.72 - 3.90] | [1.64 - 2.61] |
| Age 30-34 | 1.53 | 1.29 | 0.88 | 1.13 | 1.15 | 1.98 | 2.93 | 0.85 | 2.37 | 3.29 | 2.39 |
|  | [0.62 - 3.74] | [0.41 - 4.13] | [0.40 - 1.93] | [0.55 - 2.35] | [0.32 - 4.13] | [0.45 - 8.61] | [1.52 - 5.65] | [0.09 - 7.84] | [1.37 - 4.09] | [2.13 - 5.07] | [1.88 - 3.05] |
| Age 35-39 | 1.09 | 0.67 | 0.37 | 0.85 | 1.84 | 1.48 | 3.14 | 0.79 | 2.61 | 2.84 | 2.12 |
|  | [0.44 - 2.74] | [0.20 - 2.26] | [0.15 - 0.94] | [0.33 - 2.23] | [0.51 - 6.58] | [0.41 - 5.36] | [1.70 - 5.83] | [0.08 - 8.36] | [1.55 - 4.39] | [1.89 - 4.27] | [1.68 - 2.68] |
| Age 40-44 | 0.79 | 1.08 | 0.34 | 1.28 | 1.38 | 1.02 | 2.62 | 0.62 | 1.60 | 1.99 | 1.63 |
|  | [0.28 - 2.26] | [0.32 - 3.62] | [0.13 - 0.85] | [0.50 - 3.29] | [0.32 - 5.95] | [0.18 - 5.78] | [1.23 - 5.60] | [0.08 - 5.06] | [0.86 - 2.96] | [1.24 - 3.19] | [1.25 - 2.14] |
| Age 45-49 | 0.62 | 0.70 | 0.25 | 0.43 | 1.43 | 1.81 | 1.92 | 0.58 | 1.25 | 1.27 | 1.18 |
|  | [0.21 - 1.86] | [0.18 - 2.64] | [0.10 - 0.63] | [0.12 - 1.57] | [0.32 - 6.31] | [0.34 - 9.49] | [0.89 - 4.17] | [0.05 - 7.32] | [0.69 - 2.28] | [0.78 - 2.05] | [0.90 - 1.56] |
| Previously vs. currently married | 1.27 | 1.40 | 8.85 | 3.56 | 1.52 | 2.26 | 2.67 | 1.89 | 2.45 | 3.09 | 3.00 |
|  | [0.76 - 2.15] | [0.81 - 2.42] | [5.95 - 13.15] | [2.14 - 5.92] | [0.81 - 2.83] | [0.77 - 6.63] | [1.86 - 3.83] | [1.05 - 3.38] | [1.95 - 3.07] | [2.55 - 3.75] | [2.70 - 3.34] |
| Rural vs. urban residence | 0.87 | 1.25 | 0.84 | 0.69 | 0.68 | 1.19 | 0.71 | 0.33 | 0.69 | 0.81 | 0.76 |
|  | [0.52 - 1.43] | [0.78 - 1.99] | [0.55 - 1.29] | [0.43 - 1.12] | [0.37 - 1.22] | [0.42 - 3.37] | [0.48 - 1.04] | [0.19 - 0.57] | [0.47 - 1.00] | [0.61 - 1.08] | [0.66 - 0.87] |
| Poorest quintile |  |  |  |  |  |  |  |  |  |  |  |
|  |  |  |  |  |  |  |  |  |  |  |  |
| 2^nd^ poorest quintile | 0.67 | 0.71 | 0.89 | 1.35 | 1.27 | 0.75 | 1.04 | 0.74 | 1.26 | 1.08 | 1.00 |
|  | [0.38 - 1.20] | [0.31 - 1.64] | [0.46 - 1.71] | [0.64 - 2.85] | [0.52 - 3.10] | [0.34 - 1.66] | [0.65 - 1.67] | [0.34 - 1.61] | [0.89 - 1.77] | [0.86 - 1.37] | [0.86 - 1.15] |
| Middle quintile | 0.50 | 1.20 | 0.92 | 1.36 | 0.88 | 0.39 | 1.75 | 0.94 | 1.59 | 1.28 | 1.16 |
|  | [0.24 - 1.05] | [0.53 - 2.69] | [0.51 - 1.66] | [0.73 - 2.52] | [0.36 - 2.13] | [0.14 - 1.11] | [1.15 - 2.67] | [0.41 - 2.16] | [1.00 - 2.52] | [1.02 - 1.61] | [1.00 - 1.34] |
| 2^nd^ richest quintile | 0.19 | 1.88 | 1.52 | 1.42 | 1.19 | 0.83 | 1.96 | 0.83 | 2.56 | 1.25 | 1.37 |
|  | [0.05 - 0.66] | [0.87 - 4.07] | [0.83 - 2.82] | [0.69 - 2.93] | [0.48 - 2.97] | [0.32 - 2.20] | [1.25 - 3.09] | [0.33 - 2.09] | [1.52 - 4.31] | [0.95 - 1.66] | [1.16 - 1.61] |
| Richest quintile | 1.00 | 0.93 | 0.78 | 1.49 | 0.91 | 0.98 | 2.04 | 0.91 | 2.91 | 0.77 | 1.15 |
|  | [0.41 - 2.44] | [0.35 - 2.50] | [0.36 - 1.69] | [0.67 - 3.32] | [0.33 - 2.47] | [0.24 - 3.92] | [1.24 - 3.37] | [0.35 - 2.34] | [1.64 - 5.15] | [0.51 - 1.17] | [0.94 - 1.41] |
| No education |  |  |  |  |  |  |  |  |  |  |  |
|  |  |  |  |  |  |  |  |  |  |  |  |
| Any primary | 0.55 | 1.37 | 0.78 | 2.43 | 1.93 | 0.73 | 1.04 | 1.28 | 1.27 | 1.22 | 1.20 |
|  | [0.30 - 0.99] | [0.80 - 2.33] | [0.50 - 1.23] | [1.00 - 5.92] | [1.13 - 3.31] | [0.28 - 1.86] | [0.78 - 1.40] | [0.69 - 2.37] | [0.89 - 1.81] | [0.86 - 1.75] | [1.04 - 1.37] |
| More than primary | 0.21 | 1.15 | 0.62 | 1.84 | 1.37 | 0.50 | 1.05 | 0.59 | 1.26 | 1.28 | 1.05 |
|  | [0.09 - 0.50] | [0.56 - 2.35] | [0.39 - 0.97] | [0.72 - 4.71] | [0.60 - 3.12] | [0.11 - 2.40] | [0.61 - 1.81] | [0.17 - 1.97] | [0.82 - 1.95] | [0.88 - 1.86] | [0.89 - 1.24] |
| No employment |  |  |  |  |  |  |  |  |  |  |  |
|  |  |  |  |  |  |  |  |  |  |  |  |
| Non-manual, non-agricultural | 0.94 | 1.36 | 1.44 | 0.96 | 1.41 | 1.45 | 1.27 | 2.47 | 0.86 | 0.90 | 1.12 |
|  | [0.59 - 1.49] | [0.84 - 2.19] | [0.92 - 2.24] | [0.61 - 1.50] | [0.79 - 2.52] | [0.73 - 2.86] | [0.84 - 1.92] | [0.83 - 7.35] | [0.66 - 1.14] | [0.73 - 1.12] | [0.99 - 1.27] |
| Manual | 0.40 | 0.36 | 0.79 | 1.28 | 1.11 | 0.31 | 1.65 | 1.92 | 0.80 | 0.89 | 0.91 |
|  | [0.14 - 1.15] | [0.05 - 2.82] | [0.47 - 1.34] | [0.55 - 2.95] | [0.14 - 8.98] | [0.03 - 2.72] | [0.80 - 3.41] | [0.50 - 7.42] | [0.44 - 1.45] | [0.63 - 1.27] | [0.73 - 1.12] |
| Agricultural |  | 0.76 | 0.66 | 0.58 | 0.43 | 0.85 | 0.79 | 1.05 | 0.66 | 0.92 | 0.72 |
|  |  | [0.30 - 1.95] | [0.40 - 1.09] | [0.37 - 0.90] | [0.21 - 0.86] | [0.42 - 1.72] | [0.60 - 1.05] | [0.55 - 2.02] | [0.52 - 0.84] | [0.73 - 1.15] | [0.64 - 0.81] |
| Christian |  |  |  |  |  |  |  |  |  |  |  |
|  |  |  |  |  |  |  |  |  |  |  |  |
| Hindu |  |  | 1.20 |  |  |  |  |  |  |  |  |
|  |  |  | [0.53 - 2.71] |  |  |  |  |  |  |  |  |
| Muslim |  |  | 0.36 | 0.37 | 1.07 | 0.73 | 1.24 | 2.42 |  | 0.95 |  |
|  |  |  | [0.12 - 1.11] | [0.15 - 0.89] | [0.57 - 2.00] | [0.24 - 2.25] | [0.91 - 1.67] | [0.86 - 6.81] |  | [0.35 - 2.61] |  |
| Other/no religion |  | 0.69 | 1.22 | 0.55 | 0.52 | 1.17 | 0.29 | 1.58 |  | 1.05 |  |
|  |  | [0.26 - 1.79] | [0.41 - 3.65] | [0.13 - 2.40] | [0.06 - 4.20] | [0.29 - 4.80] | [0.04 - 2.26] | [0.37 - 6.76] |  | [0.81 - 1.36] |  |
| >1 vs. 0-1 lifetime sexual partners | 5.83 | 4.53 | 4.13 |  | 1.58 | 3.35 |  | 3.00 | 2.88 | 2.22 |  |
|  | [2.95 - 11.54] | [2.55 - 8.04] | [2.21 - 7.72] |  | [0.78 - 3.21] | [1.69 - 6.64] |  | [1.72 - 5.23] | [2.24 - 3.71] | [1.89 - 2.62] |  |

^*^ Country-level fixed effects are included for the pooled regression, but values are not shown in this table.

**Table S5: Full adjusted regression results for the association between HIV prevalence and each measure of intimate partner violence**

|  | Dominican Republic | Haiti | India | Kenya | Liberia | Malawi | Mali | Rwanda | Zambia | Zimbabwe | Pooled ^*^ |
| --- | --- | --- | --- | --- | --- | --- | --- | --- | --- | --- | --- |
| **Any physical or sexual violence** | [reference] | [reference] | [reference] | [reference] | [reference] | [reference] | [reference] | [reference] | [reference] | [reference] | [reference] |
| **vs. neither** | 1.12 | 0.45 | 1.35 | 0.88 | 0.87 | 1.07 | 1.07 | 0.99 | 0.91 | 0.97 | 1.03 |
|  | [0.67 - 1.88] | [0.23 - 0.90] | [0.95 - 1.90] | [0.62 - 1.25] | [0.56 - 1.35] | [0.51 - 2.23] | [0.81 - 1.42] | [0.59 - 1.67] | [0.77 - 1.08] | [0.83 - 1.15] | [0.94 - 1.13] |
| Age 15-19 |  |  |  |  |  |  |  |  |  |  |  |
|  |  |  |  |  |  |  |  |  |  |  |  |
| Age 20-24 | 0.69 | 1.32 | 0.65 | 1.32 | 1.06 | 1.16 | 2.53 | 0.93 | 1.19 | 1.36 | 1.37 |
|  | [0.24 - 1.98] | [0.41 - 4.26] | [0.27 - 1.56] | [0.63 - 2.79] | [0.28 - 4.00] | [0.25 - 5.35] | [1.39 - 4.61] | [0.09 - 9.78] | [0.66 - 2.15] | [0.91 - 2.04] | [1.08 - 1.74] |
| Age 25-29 | 0.79 | 1.57 | 0.72 | 1.63 | 1.51 | 1.24 | 2.66 | 0.77 | 1.93 | 2.61 | 2.06 |
|  | [0.29 - 2.13] | [0.53 - 4.70] | [0.32 - 1.61] | [0.81 - 3.29] | [0.40 - 5.77] | [0.28 - 5.50] | [1.44 - 4.92] | [0.08 - 7.35] | [1.15 - 3.25] | [1.73 - 3.93] | [1.64 - 2.60] |
| Age 30-34 | 1.53 | 1.32 | 0.84 | 1.12 | 1.13 | 1.93 | 2.93 | 0.85 | 2.40 | 3.30 | 2.39 |
|  | [0.62 - 3.74] | [0.41 - 4.18] | [0.38 - 1.87] | [0.54 - 2.31] | [0.32 - 4.02] | [0.44 - 8.44] | [1.52 - 5.64] | [0.09 - 7.85] | [1.39 - 4.16] | [2.14 - 5.10] | [1.88 - 3.04] |
| Age 35-39 | 1.10 | 0.66 | 0.36 | 0.85 | 1.81 | 1.44 | 3.15 | 0.79 | 2.63 | 2.87 | 2.12 |
|  | [0.44 - 2.75] | [0.19 - 2.26] | [0.14 - 0.90] | [0.33 - 2.20] | [0.51 - 6.36] | [0.39 - 5.24] | [1.70 - 5.85] | [0.07 - 8.44] | [1.57 - 4.43] | [1.90 - 4.31] | [1.68 - 2.69] |
| Age 40-44 | 0.80 | 1.08 | 0.32 | 1.27 | 1.34 | 0.99 | 2.64 | 0.62 | 1.61 | 2.00 | 1.64 |
|  | [0.28 - 2.28] | [0.32 - 3.62] | [0.13 - 0.82] | [0.49 - 3.28] | [0.31 - 5.69] | [0.17 - 5.63] | [1.23 - 5.64] | [0.07 - 5.08] | [0.86 - 3.00] | [1.24 - 3.22] | [1.25 - 2.14] |
| Age 45-49 | 0.62 | 0.70 | 0.24 | 0.43 | 1.40 | 1.74 | 1.94 | 0.58 | 1.25 | 1.27 | 1.18 |
|  | [0.21 - 1.87] | [0.18 - 2.63] | [0.10 - 0.61] | [0.12 - 1.55] | [0.33 - 6.03] | [0.33 - 9.09] | [0.89 - 4.19] | [0.05 - 7.30] | [0.69 - 2.29] | [0.78 - 2.07] | [0.90 - 1.56] |
| Previously vs. currently married | 1.26 | 1.44 | 8.80 | 3.40 | 1.53 | 2.31 | 2.64 | 1.89 | 2.47 | 3.09 | 2.99 |
|  | [0.75 - 2.11] | [0.83 - 2.49] | [5.93 - 13.05] | [2.07 - 5.60] | [0.83 - 2.84] | [0.76 - 6.97] | [1.83 - 3.81] | [1.06 - 3.36] | [1.97 - 3.10] | [2.54 - 3.74] | [2.69 - 3.32] |
| Rural vs. urban residence | 0.87 | 1.26 | 0.86 | 0.69 | 0.67 | 1.19 | 0.71 | 0.33 | 0.69 | 0.81 | 0.76 |
|  | [0.53 - 1.44] | [0.78 - 2.02] | [0.56 - 1.32] | [0.43 - 1.11] | [0.37 - 1.22] | [0.42 - 3.34] | [0.48 - 1.05] | [0.19 - 0.58] | [0.48 - 0.99] | [0.61 - 1.07] | [0.66 - 0.88] |
| Poorest quintile |  |  |  |  |  |  |  |  |  |  |  |
|  |  |  |  |  |  |  |  |  |  |  |  |
| 2^nd^ poorest quintile | 0.67 | 0.71 | 0.89 | 1.36 | 1.26 | 0.77 | 1.04 | 0.74 | 1.26 | 1.09 | 1.00 |
|  | [0.38 - 1.20] | [0.31 - 1.63] | [0.46 - 1.73] | [0.65 - 2.86] | [0.51 - 3.09] | [0.34 - 1.72] | [0.64 - 1.67] | [0.34 - 1.62] | [0.89 - 1.78] | [0.86 - 1.37] | [0.86 - 1.15] |
| Middle quintile | 0.51 | 1.21 | 0.93 | 1.37 | 0.88 | 0.39 | 1.75 | 0.94 | 1.59 | 1.28 | 1.16 |
|  | [0.24 - 1.06] | [0.54 - 2.73] | [0.51 - 1.68] | [0.75 - 2.52] | [0.36 - 2.13] | [0.14 - 1.12] | [1.15 - 2.66] | [0.41 - 2.16] | [1.00 - 2.53] | [1.02 - 1.61] | [1.00 - 1.34] |
| 2^nd^ richest quintile | 0.19 | 1.91 | 1.56 | 1.44 | 1.19 | 0.85 | 1.96 | 0.83 | 2.58 | 1.25 | 1.37 |
|  | [0.05 - 0.66] | [0.88 - 4.16] | [0.84 - 2.89] | [0.70 - 2.95] | [0.47 - 2.99] | [0.32 - 2.27] | [1.24 - 3.09] | [0.33 - 2.10] | [1.53 - 4.36] | [0.95 - 1.65] | [1.16 - 1.61] |
| Richest quintile | 1.01 | 0.95 | 0.82 | 1.54 | 0.90 | 1.00 | 2.03 | 0.90 | 2.92 | 0.77 | 1.15 |
|  | [0.42 - 2.46] | [0.35 - 2.54] | [0.38 - 1.81] | [0.70 - 3.42] | [0.33 - 2.46] | [0.25 - 4.03] | [1.23 - 3.36] | [0.35 - 2.35] | [1.65 - 5.17] | [0.51 - 1.17] | [0.94 - 1.41] |
| No education |  |  |  |  |  |  |  |  |  |  |  |
|  |  |  |  |  |  |  |  |  |  |  |  |
| Any primary | 0.54 | 1.36 | 0.78 | 2.40 | 1.92 | 0.74 | 1.04 | 1.28 | 1.28 | 1.23 | 1.19 |
|  | [0.30 - 0.98] | [0.80 - 2.30] | [0.50 - 1.23] | [0.99 - 5.84] | [1.12 - 3.30] | [0.30 - 1.86] | [0.78 - 1.39] | [0.69 - 2.37] | [0.90 - 1.83] | [0.86 - 1.75] | [1.04 - 1.37] |
| More than primary | 0.21 | 1.14 | 0.63 | 1.82 | 1.37 | 0.50 | 1.05 | 0.59 | 1.27 | 1.28 | 1.05 |
|  | [0.09 - 0.50] | [0.56 - 2.34] | [0.40 - 0.99] | [0.71 - 4.65] | [0.60 - 3.11] | [0.11 - 2.36] | [0.61 - 1.81] | [0.18 - 1.96] | [0.82 - 1.96] | [0.88 - 1.86] | [0.89 - 1.24] |
| No employment |  |  |  |  |  |  |  |  |  |  |  |
|  |  |  |  |  |  |  |  |  |  |  |  |
| Non-manual, non-agricultural | 0.93 | 1.36 | 1.43 | 0.92 | 1.41 | 1.47 | 1.27 | 2.47 | 0.87 | 0.90 | 1.12 |
|  | [0.59 - 1.48] | [0.84 - 2.18] | [0.92 - 2.22] | [0.59 - 1.44] | [0.80 - 2.51] | [0.74 - 2.92] | [0.84 - 1.91] | [0.85 - 7.14] | [0.66 - 1.15] | [0.72 - 1.11] | [0.99 - 1.26] |
| Manual | 0.40 | 0.36 | 0.78 | 1.26 | 1.15 | 0.31 | 1.65 | 1.92 | 0.81 | 0.88 | 0.90 |
|  | [0.14 - 1.15] | [0.05 - 2.76] | [0.46 - 1.33] | [0.55 - 2.85] | [0.15 - 9.06] | [0.03 - 2.72] | [0.80 - 3.42] | [0.50 - 7.47] | [0.44 - 1.49] | [0.62 - 1.25] | [0.73 - 1.12] |
| Agricultural |  | 0.76 | 0.66 | 0.56 | 0.42 | 0.87 | 0.79 | 1.05 | 0.67 | 0.91 | 0.72 |
|  |  | [0.30 - 1.95] | [0.40 - 1.08] | [0.36 - 0.89] | [0.21 - 0.86] | [0.43 - 1.76] | [0.60 - 1.04] | [0.55 - 2.02] | [0.52 - 0.85] | [0.73 - 1.14] | [0.64 - 0.81] |
| Christian |  |  |  |  |  |  |  |  |  |  |  |
|  |  |  |  |  |  |  |  |  |  |  |  |
| Hindu |  |  | 1.18 |  |  |  |  |  |  |  |  |
|  |  |  | [0.52 - 2.68] |  |  |  |  |  |  |  |  |
| Muslim |  |  | 0.36 | 0.37 | 1.05 | 0.73 | 1.24 | 2.42 |  | 0.96 |  |
|  |  |  | [0.12 - 1.09] | [0.15 - 0.91] | [0.55 - 1.99] | [0.23 - 2.29] | [0.92 - 1.68] | [0.87 - 6.78] |  | [0.35 - 2.63] |  |
| Other/no religion |  | 0.77 | 1.20 | 0.55 | 0.50 | 1.18 | 0.29 | 1.58 |  | 1.05 |  |
|  |  | [0.29 - 2.00] | [0.40 - 3.56] | [0.13 - 2.36] | [0.06 - 3.99] | [0.29 - 4.87] | [0.04 - 2.23] | [0.37 - 6.76] |  | [0.81 - 1.36] |  |
| >1 vs. 0-1 lifetime sexual partners | 5.79 | 4.60 | 4.14 |  | 1.59 | 3.36 |  | 3.00 | 2.89 | 2.23 |  |
|  | [2.94 - 11.41] | [2.58 - 8.18] | [2.24 - 7.65] |  | [0.79 - 3.22] | [1.70 - 6.64] |  | [1.71 - 5.27] | [2.25 - 3.72] | [1.89 - 2.63] |  |

^*^ Country-level fixed effects are included for the pooled regression, but values are not shown in this table.

**Table S5: Full adjusted regression results for the association between HIV prevalence and each measure of intimate partner violence**

|  | Dominican Republic | Haiti | India | Kenya | Liberia | Malawi | Mali | Rwanda | Zambia | Zimbabwe | Pooled ^*^ |
| --- | --- | --- | --- | --- | --- | --- | --- | --- | --- | --- | --- |
| **No sexual violence** | [reference] | [reference] | [reference] | [reference] | [reference] | [reference] | [reference] | [reference] | [reference] | [reference] | [reference] |
| **Sexual without physical violence** | 1.11 | 0.48 | 1.35 | 1.01 | 0.90 | 1.01 | 1.09 | 0.97 | 0.87 | 0.98 | 1.02 |
|  | [0.62 - 2.00] | [0.22 - 1.04] | [0.94 - 1.94] | [0.70 - 1.44] | [0.55 - 1.48] | [0.48 - 2.12] | [0.80 - 1.47] | [0.48 - 1.92] | [0.72 - 1.06] | [0.82 - 1.16] | [0.93 - 1.13] |
| **Physical and sexual violence** | 1.14 | 0.41 | 1.34 | 0.54 | 0.68 | 1.41 | 1.01 | 1.04 | 1.01 | 0.97 | 1.05 |
|  | [0.47 - 2.78] | [0.12 - 1.36] | [0.73 - 2.44] | [0.27 - 1.06] | [0.24 - 1.93] | [0.26 - 7.77] | [0.62 - 1.65] | [0.56 - 1.93] | [0.77 - 1.33] | [0.72 - 1.31] | [0.90 - 1.22] |
| Age 15-19 |  |  |  |  |  |  |  |  |  |  |  |
|  |  |  |  |  |  |  |  |  |  |  |  |
| Age 20-24 | 0.70 | 1.32 | 0.65 | 1.34 | 1.07 | 1.16 | 2.53 | 0.93 | 1.19 | 1.36 | 1.37 |
|  | [0.24 - 1.98] | [0.41 - 4.25] | [0.27 - 1.57] | [0.63 - 2.84] | [0.29 - 4.01] | [0.25 - 5.35] | [1.39 - 4.62] | [0.09 - 9.76] | [0.66 - 2.15] | [0.91 - 2.04] | [1.08 - 1.74] |
| Age 25-29 | 0.79 | 1.57 | 0.72 | 1.65 | 1.53 | 1.25 | 2.66 | 0.77 | 1.93 | 2.61 | 2.06 |
|  | [0.29 - 2.13] | [0.53 - 4.70] | [0.32 - 1.62] | [0.81 - 3.36] | [0.40 - 5.82] | [0.28 - 5.49] | [1.44 - 4.93] | [0.08 - 7.33] | [1.15 - 3.24] | [1.73 - 3.92] | [1.64 - 2.60] |
| Age 30-34 | 1.53 | 1.31 | 0.84 | 1.14 | 1.14 | 1.94 | 2.93 | 0.85 | 2.39 | 3.30 | 2.39 |
|  | [0.62 - 3.75] | [0.41 - 4.18] | [0.38 - 1.88] | [0.55 - 2.35] | [0.32 - 4.06] | [0.45 - 8.39] | [1.52 - 5.66] | [0.09 - 7.84] | [1.38 - 4.14] | [2.14 - 5.09] | [1.88 - 3.04] |
| Age 35-39 | 1.10 | 0.66 | 0.36 | 0.86 | 1.81 | 1.45 | 3.16 | 0.80 | 2.61 | 2.86 | 2.12 |
|  | [0.44 - 2.75] | [0.19 - 2.26] | [0.14 - 0.90] | [0.33 - 2.25] | [0.51 - 6.39] | [0.40 - 5.23] | [1.70 - 5.87] | [0.08 - 8.43] | [1.55 - 4.40] | [1.91 - 4.30] | [1.68 - 2.69] |
| Age 40-44 | 0.80 | 1.08 | 0.32 | 1.28 | 1.34 | 1.00 | 2.64 | 0.62 | 1.62 | 2.00 | 1.64 |
|  | [0.28 - 2.28] | [0.32 - 3.62] | [0.13 - 0.83] | [0.50 - 3.30] | [0.32 - 5.73] | [0.18 - 5.63] | [1.23 - 5.64] | [0.08 - 5.07] | [0.87 - 3.01] | [1.25 - 3.21] | [1.25 - 2.14] |
| Age 45-49 | 0.62 | 0.69 | 0.24 | 0.44 | 1.41 | 1.77 | 1.94 | 0.58 | 1.25 | 1.27 | 1.18 |
|  | [0.21 - 1.86] | [0.18 - 2.62] | [0.10 - 0.61] | [0.12 - 1.59] | [0.33 - 6.07] | [0.34 - 9.22] | [0.90 - 4.22] | [0.05 - 7.29] | [0.69 - 2.28] | [0.79 - 2.06] | [0.90 - 1.56] |
| Previously vs. currently married | 1.26 | 1.44 | 8.80 | 3.63 | 1.55 | 2.28 | 2.64 | 1.88 | 2.44 | 3.09 | 2.99 |
|  | [0.74 - 2.13] | [0.84 - 2.49] | [5.90 - 13.13] | [2.18 - 6.04] | [0.83 - 2.87] | [0.74 - 6.97] | [1.83 - 3.83] | [1.06 - 3.34] | [1.94 - 3.07] | [2.55 - 3.74] | [2.68 - 3.32] |
| Rural vs. urban residence | 0.87 | 1.26 | 0.86 | 0.69 | 0.67 | 1.19 | 0.71 | 0.33 | 0.69 | 0.81 | 0.76 |
|  | [0.53 - 1.44] | [0.78 - 2.01] | [0.56 - 1.32] | [0.43 - 1.12] | [0.37 - 1.22] | [0.42 - 3.35] | [0.48 - 1.05] | [0.19 - 0.58] | [0.48 - 0.99] | [0.61 - 1.07] | [0.66 - 0.88] |
| Poorest quintile |  |  |  |  |  |  |  |  |  |  |  |
|  |  |  |  |  |  |  |  |  |  |  |  |
| 2^nd^ poorest quintile | 0.67 | 0.71 | 0.89 | 1.34 | 1.27 | 0.76 | 1.04 | 0.74 | 1.26 | 1.09 | 0.99 |
|  | [0.38 - 1.20] | [0.31 - 1.63] | [0.46 - 1.73] | [0.64 - 2.83] | [0.52 - 3.09] | [0.34 - 1.69] | [0.64 - 1.68] | [0.34 - 1.62] | [0.89 - 1.77] | [0.86 - 1.37] | [0.86 - 1.15] |
| Middle quintile | 0.51 | 1.21 | 0.93 | 1.34 | 0.88 | 0.39 | 1.75 | 0.94 | 1.58 | 1.28 | 1.16 |
|  | [0.24 - 1.06] | [0.54 - 2.72] | [0.51 - 1.69] | [0.73 - 2.49] | [0.36 - 2.13] | [0.14 - 1.11] | [1.15 - 2.67] | [0.41 - 2.15] | [1.00 - 2.52] | [1.02 - 1.61] | [1.00 - 1.34] |
| 2^nd^ richest quintile | 0.19 | 1.91 | 1.56 | 1.39 | 1.19 | 0.84 | 1.96 | 0.83 | 2.57 | 1.25 | 1.37 |
|  | [0.05 - 0.66] | [0.88 - 4.16] | [0.84 - 2.89] | [0.68 - 2.87] | [0.48 - 2.98] | [0.31 - 2.26] | [1.24 - 3.09] | [0.33 - 2.10] | [1.53 - 4.34] | [0.95 - 1.65] | [1.16 - 1.61] |
| Richest quintile | 1.01 | 0.94 | 0.82 | 1.48 | 0.90 | 0.98 | 2.04 | 0.90 | 2.91 | 0.77 | 1.15 |
|  | [0.42 - 2.47] | [0.35 - 2.53] | [0.38 - 1.81] | [0.66 - 3.30] | [0.33 - 2.47] | [0.24 - 3.96] | [1.23 - 3.36] | [0.34 - 2.35] | [1.65 - 5.16] | [0.51 - 1.17] | [0.94 - 1.41] |
| No education |  |  |  |  |  |  |  |  |  |  |  |
|  |  |  |  |  |  |  |  |  |  |  |  |
| Any primary | 0.54 | 1.36 | 0.78 | 2.43 | 1.93 | 0.73 | 1.04 | 1.28 | 1.28 | 1.23 | 1.19 |
|  | [0.30 - 0.98] | [0.80 - 2.30] | [0.50 - 1.22] | [1.00 - 5.91] | [1.12 - 3.31] | [0.29 - 1.87] | [0.78 - 1.40] | [0.69 - 2.37] | [0.90 - 1.82] | [0.86 - 1.75] | [1.04 - 1.37] |
| More than primary | 0.22 | 1.14 | 0.63 | 1.85 | 1.37 | 0.50 | 1.05 | 0.59 | 1.27 | 1.28 | 1.05 |
|  | [0.09 - 0.50] | [0.56 - 2.33] | [0.40 - 0.99] | [0.72 - 4.70] | [0.60 - 3.12] | [0.11 - 2.38] | [0.61 - 1.81] | [0.18 - 2.00] | [0.82 - 1.96] | [0.88 - 1.86] | [0.89 - 1.24] |
| No employment |  |  |  |  |  |  |  |  |  |  |  |
|  |  |  |  |  |  |  |  |  |  |  |  |
| Non-manual, non-agricultural | 0.93 | 1.36 | 1.43 | 0.95 | 1.41 | 1.45 | 1.27 | 2.46 | 0.87 | 0.90 | 1.12 |
|  | [0.59 - 1.48] | [0.85 - 2.18] | [0.91 - 2.22] | [0.61 - 1.48] | [0.79 - 2.51] | [0.73 - 2.88] | [0.84 - 1.91] | [0.85 - 7.17] | [0.66 - 1.14] | [0.72 - 1.11] | [0.99 - 1.26] |
| Manual | 0.40 | 0.36 | 0.78 | 1.27 | 1.13 | 0.31 | 1.65 | 1.91 | 0.81 | 0.89 | 0.90 |
|  | [0.14 - 1.15] | [0.05 - 2.77] | [0.46 - 1.33] | [0.55 - 2.91] | [0.14 - 8.90] | [0.03 - 2.73] | [0.80 - 3.43] | [0.50 - 7.27] | [0.44 - 1.48] | [0.62 - 1.26] | [0.73 - 1.12] |
| Agricultural |  | 0.76 | 0.66 | 0.57 | 0.42 | 0.86 | 0.79 | 1.05 | 0.66 | 0.91 | 0.72 |
|  |  | [0.30 - 1.95] | [0.40 - 1.08] | [0.36 - 0.89] | [0.21 - 0.86] | [0.43 - 1.75] | [0.60 - 1.04] | [0.55 - 2.01] | [0.52 - 0.85] | [0.73 - 1.14] | [0.64 - 0.81] |
| Christian |  |  |  |  |  |  |  |  |  |  |  |
|  |  |  |  |  |  |  |  |  |  |  |  |
| Hindu |  |  | 1.18 |  |  |  |  |  |  |  |  |
|  |  |  | [0.52 - 2.68] |  |  |  |  |  |  |  |  |
| Muslim |  |  | 0.36 | 0.36 | 1.06 | 0.73 | 1.24 | 2.40 |  | 0.96 |  |
|  |  |  | [0.12 - 1.09] | [0.15 - 0.89] | [0.56 - 2.00] | [0.24 - 2.25] | [0.92 - 1.68] | [0.85 - 6.82] |  | [0.35 - 2.63] |  |
| Other/no religion |  | 0.76 | 1.20 | 0.54 | 0.51 | 1.19 | 0.29 | 1.58 |  | 1.05 |  |
|  |  | [0.29 - 1.98] | [0.40 - 3.55] | [0.12 - 2.36] | [0.06 - 4.06] | [0.29 - 4.82] | [0.04 - 2.26] | [0.37 - 6.73] |  | [0.81 - 1.36] |  |
| >1 vs. 0-1 lifetime sexual partners | 5.79 | 4.60 | 4.14 |  | 1.59 | 3.36 |  | 3.00 | 2.88 | 2.23 |  |
|  | [2.94 - 11.41] | [2.59 - 8.19] | [2.22 - 7.71] |  | [0.78 - 3.22] | [1.71 - 6.63] |  | [1.70 - 5.29] | [2.24 - 3.71] | [1.89 - 2.63] |  |

^*^ Country-level fixed effects are included for the pooled regression, but values are not shown in this table.
