# Supplementary material for: No Association between HIV and Intimate Partner Violence among Women in 10 Developing Countries
Source: PLoS One. 2010 Dec 8;5(12):e14257. doi: 10.1371/journal.pone.0014257 (PMC2999537; doi:10.1371/journal.pone.0014257)
Supplement: Table S6 — Unadjusted odds ratios [95% confidence intervals] for HIV prevalence and intimate partner violence by different sample weightings (0.07 MB DOC) [file pone.0014257.s006.doc]

**Table S6: Unadjusted odds ratios [95% confidence intervals] for HIV prevalence and intimate partner violence by different sample weightings**

|  | **Dominican Republic** | **Haiti** | **India** | **Kenya** | **Liberia** | **Malawi** | **Mali** | **Rwanda** | **Zambia** | **Zimbabwe** | **Pooled *** |
| --- | --- | --- | --- | --- | --- | --- | --- | --- | --- | --- | --- |
| **Physical or sexual violence vs. neither** |  |  |  |  |  |  |  |  |  |  |  |
| No weights | 1.43 | 0.54 | 1.69 | 0.97 | 1.00 | 1.31 | 1.11 | 1.22 | 1.13 | 1.02 | 1.10 |
|  | [0.87 - 2.36] | [0.28 - 1.05] | [1.23 - 2.34] | [0.69 - 1.37] | [0.64 - 1.57] | [0.68 - 2.53] | [0.85 - 1.45] | [0.76 - 1.95] | [0.98 - 1.31] | [0.88 - 1.19] | [1.01 - 1.19] |
| HIV weights | 1.67 | 0.63 | 1.37 | 1.06 | 0.99 | 1.07 | 1.17 | 1.17 | 1.20 | 1.07 | 1.13 |
|  | [0.80 - 3.47] | [0.28 - 1.41] | [0.83 - 2.28] | [0.72 - 1.55] | [0.56 - 1.75] | [0.52 - 2.19] | [0.86 - 1.60] | [0.73 - 1.88] | [1.00 - 1.45] | [0.92 - 1.24] | [1.02 - 1.24] |
| IPV weights | 1.84 | 0.53 | 1.33 | 1.04 | 0.78 | 1.07 | 1.18 | 1.32 | 1.18 | 1.08 | 1.13 |
|  | [0.90 - 3.77] | [0.23 - 1.23] | [0.83 - 2.14] | [0.69 - 1.57] | [0.44 - 1.39] | [0.52 - 2.20] | [0.85 - 1.63] | [0.82 - 2.13] | [0.98 - 1.44] | [0.90 - 1.31] | [1.01 - 1.25] |
| HIV & IPV weights | 2.34 | 0.56 | 1.39 | 1.13 | 0.69 | 0.54 | 1.34 | 1.26 | 1.24 | 1.09 | 1.16 |
|  | [0.74 - 7.41] | [0.18 - 1.68] | [0.76 - 2.55] | [0.71 - 1.79] | [0.32 - 1.52] | [0.19 - 1.51] | [0.83 - 2.16] | [0.74 - 2.15] | [0.98 - 1.58] | [0.89 - 1.34] | [1.01 - 1.34] |
|  |  |  |  |  |  |  |  |  |  |  |  |
| **Sexual without physical violence vs. no sexual violence** |  |  |  |  |  |  |  |  |  |  |  |
| No weights | 1.36 | 0.54 | 1.59 | 1.08 | 1.05 | 1.22 | 1.12 | 1.05 | 1.01 | 1.02 | 1.06 |
|  | [0.75 - 2.44] | [0.25 - 1.16] | [1.13 - 2.24] | [0.76 - 1.53] | [0.63 - 1.74] | [0.60 - 2.49] | [0.84 - 1.50] | [0.56 - 1.97] | [0.86 - 1.20] | [0.86 - 1.21] | [0.97 - 1.17] |
| HIV weights | 1.87 | 0.75 | 0.97 | 1.15 | 1.05 | 1.05 | 1.18 | 1.03 | 1.08 | 1.05 | 1.08 |
|  | [0.82 - 4.23] | [0.30 - 1.86] | [0.59 - 1.59] | [0.79 - 1.68] | [0.56 - 1.95] | [0.47 - 2.30] | [0.83 - 1.67] | [0.52 - 2.04] | [0.90 - 1.30] | [0.88 - 1.25] | [0.97 - 1.20] |
| IPV weights | 2.02 | 0.60 | 1.24 | 1.15 | 0.86 | 1.06 | 1.17 | 1.17 | 1.04 | 1.07 | 1.08 |
|  | [0.91 - 4.50] | [0.23 - 1.55] | [0.73 - 2.09] | [0.76 - 1.72] | [0.46 - 1.60] | [0.48 - 2.32] | [0.84 - 1.64] | [0.61 - 2.24] | [0.84 - 1.29] | [0.87 - 1.32] | [0.96 - 1.22] |
| HIV & IPV weights | 2.77 | 0.68 | 1.05 | 1.23 | 0.79 | 0.67 | 1.23 | 1.13 | 1.08 | 1.03 | 1.09 |
|  | [0.80 - 9.56] | [0.21 - 2.23] | [0.57 - 1.92] | [0.78 - 1.92] | [0.34 - 1.84] | [0.25 - 1.78] | [0.76 - 2.00] | [0.55 - 2.32] | [0.84 - 1.39] | [0.82 - 1.30] | [0.94 - 1.27] |
|  |  |  |  |  |  |  |  |  |  |  |  |
| **Physical & sexual violence vs. no sexual violence** |  |  |  |  |  |  |  |  |  |  |  |
| No weights | 1.62 | 0.56 | 2.24 | 0.68 | 0.77 | 1.82 | 1.07 | 1.64 | 1.46 | 1.03 | 1.20 |
|  | [0.70 - 3.74] | [0.17 - 1.81] | [1.29 - 3.89] | [0.34 - 1.35] | [0.27 - 2.24] | [0.39 - 8.36] | [0.67 - 1.72] | [0.90 - 2.98] | [1.16 - 1.83] | [0.79 - 1.35] | [1.04 - 1.39] |
| HIV weights | 1.09 | 0.36 | 2.90 | 0.83 | 0.69 | 1.18 | 1.16 | 1.53 | 1.55 | 1.13 | 1.27 |
|  | [0.32 - 3.63] | [0.09 - 1.40] | [1.24 - 6.79] | [0.40 - 1.71] | [0.22 - 2.21] | [0.24 - 5.74] | [0.65 - 2.08] | [0.81 - 2.90] | [1.18 - 2.04] | [0.82 - 1.55] | [1.07 - 1.51] |
| IPV weights | 1.32 | 0.35 | 1.83 | 0.78 | 0.46 | 1.11 | 1.20 | 1.69 | 1.59 | 1.12 | 1.26 |
|  | [0.41 - 4.25] | [0.10 - 1.25] | [0.87 - 3.85] | [0.36 - 1.70] | [0.14 - 1.55] | [0.23 - 5.35] | [0.65 - 2.22] | [0.87 - 3.28] | [1.18 - 2.14] | [0.78 - 1.61] | [1.05 - 1.51] |
| HIV & IPV weights | 0.92 | 0.18 | 2.64 | 0.89 | 0.32 | 0.26 | 1.75 | 1.58 | 1.69 | 1.29 | 1.38 |
|  | [0.20 - 4.16] | [0.03 - 0.97] | [0.93 - 7.48] | [0.39 - 2.06] | [0.08 - 1.34] | [0.04 - 1.77] | [0.67 - 4.58] | [0.76 - 3.27] | [1.18 - 2.43] | [0.88 - 1.90] | [1.10 - 1.73] |

^*^ Country-level fixed effects were included in the pooled regressions, but are not shown in this table.
