# Supplementary material for: No Association between HIV and Intimate Partner Violence among Women in 10 Developing Countries
Source: PLoS One. 2010 Dec 8;5(12):e14257. doi: 10.1371/journal.pone.0014257 (PMC2999537; doi:10.1371/journal.pone.0014257)
Supplement: Table S7 — Comparison of main results with and without the Indian sample (0.10 MB DOC) [file pone.0014257.s007.doc]

**Table S7: Comparison of main results with and without the Indian sample**

|  | **With India (n=60,114)** | |  | **Without India (n=30,331)** | |  | **India (n=29,783)** | |
| --- | --- | --- | --- | --- | --- | --- | --- | --- |
| **Univariate analysis** |  |  |  |  |  |  |  |  |
|  |  |  |  |  |  |  |  |  |
| Physical or sexual violence | 1.10 |  |  | 1.06 |  |  | 1.69 |  |
|  | [1.01 - 1.19] |  |  | [0.97 - 1.16] |  |  | [1.23 - 2.34] |  |
| Sexual without physical violence |  | 1.06 |  |  | 1.03 |  |  | 1.59 |
|  |  | [0.97 - 1.17] |  |  | [0.93 - 1.14] |  |  | [1.13 - 2.24] |
| Physical & sexual violence |  | 1.20 |  |  | 1.16 |  |  | 2.24 |
|  |  | [1.04 - 1.39] |  |  | [1.00 - 1.34] |  |  | [1.29 - 3.89] |
|  |  |  |  |  |  |  |  |  |
|  |  |  |  |  |  |  |  |  |
| **Multivariate analysis** |  |  |  |  |  |  |  |  |
|  |  |  |  |  |  |  |  |  |
| Physical or sexual violence | 1.03 |  |  | 1.00 |  |  | 1.38 |  |
|  | [0.94 - 1.13] |  |  | [0.91 - 1.10] |  |  | [0.97 - 1.95] |  |
| Physical without sexual violence |  | 1.02 |  |  | 0.99 |  |  | 1.36 |
|  |  | [0.93 - 1.13] |  |  | [0.90 - 1.10] |  |  | [0.94 - 1.96] |
| Physical & sexual violence |  | 1.05 |  |  | 1.02 |  |  | 1.45 |
|  |  | [0.90 - 1.22] |  |  | [0.87 - 1.19] |  |  | [0.81 - 2.60] |
| Age 15-19 |  |  |  |  |  |  |  |  |
|  |  |  |  |  |  |  |  |  |
| Age 20-24 | 1.37 | 1.37 |  | 1.44 | 1.44 |  | 0.68 | 0.68 |
|  | [1.08 - 1.74] | [1.08 - 1.74] |  | [1.12 - 1.84] | [1.12 - 1.84] |  | [0.29 - 1.63] | [0.29 - 1.63] |
| Age 25-29 | 2.06 | 2.06 |  | 2.19 | 2.19 |  | 0.77 | 0.77 |
|  | [1.64 - 2.60] | [1.64 - 2.60] |  | [1.73 - 2.79] | [1.73 - 2.79] |  | [0.34 - 1.71] | [0.34 - 1.72] |
| Age 30-34 | 2.39 | 2.39 |  | 2.53 | 2.53 |  | 0.91 | 0.91 |
|  | [1.88 - 3.04] | [1.88 - 3.04] |  | [1.97 - 3.25] | [1.97 - 3.25] |  | [0.41 - 2.02] | [0.41 - 2.03] |
| Age 35-39 | 2.12 | 2.12 |  | 2.37 | 2.37 |  | 0.38 | 0.39 |
|  | [1.68 - 2.69] | [1.68 - 2.69] |  | [1.87 - 3.01] | [1.87 - 3.01] |  | [0.15 - 0.97] | [0.15 - 0.98] |
| Age 40-44 | 1.64 | 1.64 |  | 1.83 | 1.83 |  | 0.35 | 0.35 |
|  | [1.25 - 2.14] | [1.25 - 2.14] |  | [1.39 - 2.41] | [1.39 - 2.41] |  | [0.14 - 0.88] | [0.14 - 0.89] |
| Age 45-49 | 1.18 | 1.18 |  | 1.32 | 1.32 |  | 0.26 | 0.26 |
|  | [0.90 - 1.56] | [0.90 - 1.56] |  | [0.99 - 1.75] | [0.99 - 1.75] |  | [0.10 - 0.65] | [0.10 - 0.65] |
| Previously vs. currently married | 2.99 | 2.99 |  | 2.76 | 2.75 |  | 9.24 | 9.21 |
|  | [2.69 - 3.32] | [2.68 - 3.32] |  | [2.48 - 3.07] | [2.47 - 3.07] |  | [6.29 - 13.59] | [6.23 - 13.61] |
| Rural vs. urban residence | 0.76 | 0.76 |  | 0.76 | 0.76 |  | 0.92 | 0.92 |
|  | [0.66 - 0.88] | [0.66 - 0.88] |  | [0.65 - 0.88] | [0.65 - 0.88] |  | [0.60 - 1.41] | [0.60 - 1.41] |
| Poorest quintile |  |  |  |  |  |  |  |  |
|  |  |  |  |  |  |  |  |  |
| 2nd poorest quintile | 1.00 | 0.99 |  | 0.99 | 0.99 |  | 0.89 | 0.89 |
|  | [0.86 - 1.15] | [0.86 - 1.15] |  | [0.86 - 1.16] | [0.86 - 1.15] |  | [0.46 - 1.70] | [0.46 - 1.70] |
| Middle quintile | 1.16 | 1.16 |  | 1.16 | 1.16 |  | 0.92 | 0.92 |
|  | [1.00 - 1.34] | [1.00 - 1.34] |  | [1.00 - 1.35] | [1.00 - 1.35] |  | [0.51 - 1.67] | [0.51 - 1.67] |
| 2nd richest quintile | 1.37 | 1.37 |  | 1.35 | 1.35 |  | 1.49 | 1.49 |
|  | [1.16 - 1.61] | [1.16 - 1.61] |  | [1.14 - 1.60] | [1.14 - 1.60] |  | [0.80 - 2.77] | [0.80 - 2.77] |
| Richest quintile | 1.15 | 1.15 |  | 1.21 | 1.21 |  | 0.81 | 0.81 |
|  | [0.94 - 1.41] | [0.94 - 1.41] |  | [0.98 - 1.49] | [0.98 - 1.49] |  | [0.37 - 1.79] | [0.37 - 1.79] |
| No education |  |  |  |  |  |  |  |  |
|  |  |  |  |  |  |  |  |  |
| Any primary | 1.19 | 1.19 |  | 1.31 | 1.31 |  | 0.81 | 0.81 |
|  | [1.04 - 1.37] | [1.04 - 1.37] |  | [1.14 - 1.52] | [1.13 - 1.52] |  | [0.52 - 1.27] | [0.52 - 1.27] |
| More than primary | 1.05 | 1.05 |  | 1.19 | 1.19 |  | 0.65 | 0.65 |
|  | [0.89 - 1.24] | [0.89 - 1.24] |  | [1.00 - 1.42] | [1.00 - 1.42] |  | [0.42 - 1.00] | [0.42 - 1.00] |
| No employment |  |  |  |  |  |  |  |  |
|  |  |  |  |  |  |  |  |  |
| Non-manual, non-agricultural | 1.12 | 1.12 |  | 1.07 | 1.07 |  | 1.59 | 1.59 |
|  | [0.99 - 1.26] | [0.99 - 1.26] |  | [0.95 - 1.21] | [0.94 - 1.21] |  | [1.03 - 2.46] | [1.02 - 2.47] |
| Manual | 0.90 | 0.90 |  | 0.88 | 0.88 |  | 0.80 | 0.80 |
|  | [0.73 - 1.12] | [0.73 - 1.12] |  | [0.70 - 1.11] | [0.70 - 1.11] |  | [0.47 - 1.37] | [0.47 - 1.37] |
| Agricultural | 0.72 | 0.72 |  | 0.71 | 0.71 |  | 0.70 | 0.70 |
|  | [0.64 - 0.81] | [0.64 - 0.81] |  | [0.63 - 0.80] | [0.63 - 0.80] |  | [0.43 - 1.14] | [0.43 - 1.14] |
