# Supplementary material for: No Association between HIV and Intimate Partner Violence among Women in 10 Developing Countries
Source: PLoS One. 2010 Dec 8;5(12):e14257. doi: 10.1371/journal.pone.0014257 (PMC2999537; doi:10.1371/journal.pone.0014257)
Supplement: Table S8 — Main results for unweighted country pooled regressions stratified by marital status (0.04 MB DOC) [file pone.0014257.s008.doc]

**Table S8: Main results for unweighted country pooled regressions stratified by marital status**

|  | **Physical or sexual violence vs. neither** | |  | **Sexual without physical violence vs. no sexual violence** | |  | **Physical & sexual violence vs. no sexual violence** | |
| --- | --- | --- | --- | --- | --- | --- | --- | --- |
|  | **Unadjusted** | **Adjusted** |  | **Unadjusted** | **Adjusted** |  | **Unadjusted** | **Adjusted** |
|  |  |  |  |  |  |  |  |  |
| Full sample (n=60,114) | 1.10 | 1.03 |  | 1.03 | 1.02 |  | 1.13 | 1.05 |
|  | [1.01 - 1.19] | [0.94 - 1.13] |  | [0.92 - 1.16] | [0.93 - 1.13] |  | [0.95 - 1.35] | [0.90 - 1.22] |
|  |  |  |  |  |  |  |  |  |
| Currently married women only (n=54,203) | 1.06 | 1.07 |  | 1.03 | 1.05 |  | 1.13 | 1.15 |
|  | [0.95 - 1.17] | [0.96 - 1.19] |  | [0.92 - 1.16] | [0.93 - 1.18] |  | [0.95 - 1.35] | [0.96 - 1.37] |
|  |  |  |  |  |  |  |  |  |
| Previously married women only (n=5,911) | 0.94 | 0.94 |  | 0.96 | 0.97 |  | 0.88 | 0.87 |
|  | [0.79 - 1.11] | [0.79 - 1.12] |  | [0.79 - 1.18] | [0.79 - 1.19] |  | [0.69 - 1.14] | [0.67 - 1.12] |
|  |  |  |  |  |  |  |  |  |
