# Supplementary material for: No Association between HIV and Intimate Partner Violence among Women in 10 Developing Countries
Source: PLoS One. 2010 Dec 8;5(12):e14257. doi: 10.1371/journal.pone.0014257 (PMC2999537; doi:10.1371/journal.pone.0014257)
Supplement: Table S9 — Comparison of those refusing and not refusing HIV tests (0.12 MB DOC) [file pone.0014257.s009.doc]

**Table S9: Comparison of those refusing and not refusing HIV tests**

|  | **Dominican Republic** | |  | **India** | |  | **Liberia** | |  | **Mali** | |  | **Malawi** | |  | **Zambia** | |
| --- | --- | --- | --- | --- | --- | --- | --- | --- | --- | --- | --- | --- | --- | --- | --- | --- | --- |
| Sample, N (%) |  |  |  |  |  |  |  |  |  |  |  |  |  |  |  |  |  |
|  |  |  |  |  |  |  |  |  |  |  |  |  |  |  |  |  |  |
| Offered HIV test and decision information available | 27,411 |  |  | 57,629 |  |  | 7,065 |  |  | 4,956 |  |  | 3,826 |  |  | 7,100 |  |
| Selected for DV module | 10,416 |  |  | 84,356 |  |  | 4,995 |  |  | 10,272 |  |  | 9,863 |  |  | 5,259 |  |
| Both of the above | 10,392 |  |  | 42,041 |  |  | 4,983 |  |  | 3,400 |  |  | 3,245 |  |  | 5,232 |  |
|  |  |  |  |  |  |  |  |  |  |  |  |  |  |  |  |  |  |
| Did not respond to all three DV questions ^1^ | 1,918 | (18.5) |  | 7,535 | (17.9) |  | 1,121 | (22.5) |  | 443 | (13.0) |  | 470 | (14.5) |  | 1,038 | (19.8) |
| Responded to DV; HIV consent not requested ^2^ | 57 | (0.5) |  | 2,550 | (6.1) |  | 21 | (0.4) |  | 18 | (0.5) |  | 658 | (20.3) |  | 15 | (0.3) |
| Responded to DV; refused HIV test | 456 | (4.4) |  | 2,016 | (4.8) |  | 273 | (5.5) |  | 85 | (2.5) |  | 9 | (0.3) |  | 792 | (15.1) |
| Completed DV questions and HIV results obtained | 7,961 | (76.6) |  | 29,940 | (71.2) |  | 3,568 | (71.6) |  | 2,854 | (83.9) |  | 2,108 | (65.0) |  | 3,387 | (64.7) |
|  |  |  |  |  |  |  |  |  |  |  |  |  |  |  |  |  |  |
|  |  |  |  |  |  |  |  |  |  |  |  |  |  |  |  |  |  |
|  | **%** | **Wald (p-value)** |  | **%** | **Wald (p-value)** |  | **%** | **Wald (p-value)** |  | **%** | **Wald (p-value)** |  | **%** | **Wald (p-value)** |  | **%** | **Wald (p-value)** |
|  |  |  |  |  |  |  |  |  |  |  |  |  |  |  |  |  |  |
| Any physical abuse |  |  |  |  |  |  |  |  |  |  |  |  |  |  |  |  |  |
| Accepted HIV test | 15.71 |  |  | 34.09 |  |  | 36.24 |  |  | 16.08 |  |  | 21.44 |  |  | 44.91 |  |
| Refused HIV test | 8.11 |  |  | 25.1 |  |  | 35.16 |  |  | 18.82 |  |  | 11.11 |  |  | 39.27 |  |
| Difference | 7.60 | 4.38 |  | 9.00 | 8.28 |  | 1.07 | 0.36 |  | -2.74 | 0.68 |  | 10.33 | 0.75 |  | 5.64 | 2.88 |
|  |  | (<0.001) |  |  | (<0.001) |  |  | (0.722) |  |  | (0.499) |  |  | (0.451) |  |  | (0.004) |
| Any sexual abuse |  |  |  |  |  |  |  |  |  |  |  |  |  |  |  |  |  |
| Accepted HIV test | 5.55 |  |  | 6.89 |  |  | 9.14 |  |  | 3.47 |  |  | 13.95 |  |  | 17.09 |  |
| Refused HIV test | 2.63 |  |  | 3.47 |  |  | 10.26 |  |  | 8.24 |  |  | 0 |  |  | 13.26 |  |
| Difference | 2.92 | 2.68 |  | 3.42 | 5.96 |  | -1.12 | 0.62 |  | -4.77 | 2.32 |  | 13.95 | 1.21 |  | 3.84 | 2.63 |
|  |  | (0.007) |  |  | (<0.001) |  |  | (0.538) |  |  | (0.020) |  |  | (0.227) |  |  | (0.009) |
| Any physical or sexual abuse |  |  |  |  |  |  |  |  |  |  |  |  |  |  |  |  |  |
| Accepted HIV test | 16.59 |  |  | 35.19 |  |  | 38.96 |  |  | 17.10 |  |  | 28.04 |  |  | 48.57 |  |
| Refused HIV test | 8.77 |  |  | 25.30 |  |  | 38.46 |  |  | 22.35 |  |  | 11.11 |  |  | 41.16 |  |
| Difference | 7.82 | 4.41 |  | 9.89 | 9.04 |  | 0.5 | 0.16 |  | -5.25 | 1.26 |  | 16.92 | 1.13 |  | 7.41 | 3.76 |
|  |  | (<0.001) |  |  | (<0.001) |  |  | (0.871) |  |  | (0.210) |  |  | (0.259) |  |  | (<0.001) |
| Sexual abuse without physical abuse |  |  |  |  |  |  |  |  |  |  |  |  |  |  |  |  |  |
| Accepted HIV test | 11.92 |  |  | 29.39 |  |  | 32.54 |  |  | 14.65 |  |  | 20.68 |  |  | 35.13 |  |
| Refused HIV test | 6.8 |  |  | 22.02 |  |  | 31.50 |  |  | 17.65 |  |  | 11.11 |  |  | 29.8 |  |
| Difference | 5.12 | 3.32 |  | 7.37 | 7.06 |  | 1.04 | 0.35 |  | -3.00 | 0.77 |  | 9.57 | 0.71 |  | 5.34 | 2.85 |
|  |  | (0.001) |  |  | (<0.001) |  |  | (0.724) |  |  | (0.44) |  |  | (0.479) |  |  | (0.004) |
| Both physical and sexual abuse |  |  |  |  |  |  |  |  |  |  |  |  |  |  |  |  |  |
| Accepted HIV test | 4.67 |  |  | 5.80 |  |  | 6.42 |  |  | 2.45 |  |  | 7.35 |  |  | 13.43 |  |
| Refused HIV test | 1.97 |  |  | 3.27 |  |  | 6.96 |  |  | 4.71 |  |  | 0 |  |  | 11.36 |  |
| Difference | 2.70 | 2.70 |  | 2.52 | 4.76 |  | -0.54 | 0.35 |  | -2.25 | 1.31 |  | 7.35 | 0.85 |  | 2.07 | 1.56 |
|  |  | (0.007) |  |  | (<0.001) |  |  | (0.726) |  |  | (0.191) |  |  | (0.398) |  |  | (0.119) |

Note. DV: Domestic violence. Information on HIV test acceptance not available in Haiti, Kenya, Rwanda and Zimbabwe. Both 'consent not requested' and 'DV responses missing' were excluded from difference analyses under the assumption that this missingness was random.

^1^ DV non-responders include both those who did not complete the DV module, and those who only responded to some of the questions.

^2^ Consent not requested' includes those absent at the time of HIV test request and those for whom information on the response to HIV test consent is not recorded.
