# Supplementary material for: No Association between HIV and Intimate Partner Violence among Women in 10 Developing Countries
Source: PLoS One. 2010 Dec 8;5(12):e14257. doi: 10.1371/journal.pone.0014257 (PMC2999537; doi:10.1371/journal.pone.0014257)
Supplement: Table S10 — Adjusted odds ratios and 95% confidence intervals for the association between HIV prevalence and measures of intimate partner violence in India (0.05 MB DOC) [file pone.0014257.s010.doc]

**Table S10: Adjusted odds ratios and 95% confidence intervals for the association between HIV prevalence and measures of intimate partner violence in India**

|  | **Sample weights  used** | **Any physical violence vs. no physical violence** |  | **Physical without sexual violence vs. no physical violence** | **Physical & sexual violence  vs. no physical violence** |
| --- | --- | --- | --- | --- | --- |
| **Silverman et al.’s sample (n=28,139) ^1^** | **HIV** | 1.53 |  | 0.89 | 3.92 |
|  |  | [0.76 - 3.06] |  | [0.46 - 1.71] | [1.41 - 10.94] |
|  |  |  |  |  |  |
| **This study’s sample (n=29,783) ^2^** |  |  |  |  |  |
| **Silverman’s covariates** | **HIV** | 1.28 |  | 0.88 | 2.75 |
|  |  | [0.75 - 2.21] |  | [0.53 - 1.47] | [1.14 - 6.65] |
|  | **DV** | 1.18 |  | 1.08 | 1.67 |
|  |  | [0.71 - 1.94] |  | [0.62 - 1.87] | [0.78 - 3.60] |
|  | **HIV and DV** | 1.36 |  | 1.01 | 2.66 |
|  |  | [0.70 - 2.67] |  | [0.52 - 1.93] | [0.90 - 7.85] |
|  | **None** | 1.51 |  | 1.40 | 2.05 |
|  |  | [1.07 - 2.12] |  | [0.97 - 2.01] | [1.16 - 3.62] |
|  |  |  |  |  |  |
| **Our covariates ^3^** | **HIV** | 1.11 |  | 0.81 | 2.02 |
|  |  | [0.64 - 1.92] |  | [0.48 - 1.37] | [0.79 - 5.13] |
|  | **DV** | 1.00 |  | 0.99 | 1.05 |
|  |  | [0.61 - 1.66] |  | [0.57 - 1.71] | [0.45 - 2.47] |
|  | **HIV and DV** | 1.10 |  | 0.87 | 1.81 |
|  |  | [0.56 - 2.16] |  | [0.45 - 1.67] | [0.56 - 5.85] |
|  | **None** | 1.29 |  | 1.29 | 1.32 |
|  |  | [0.92 - 1.81] |  | [0.90 - 1.84] | [0.73 - 2.40] |

**Footnotes:**

1. Figure for Silverman et al. taken from Table 2 of their paper. Covariates used were age (5-year increments), education (none, primary or secondary), household wealth (5 quintiles), lifetime number of sex partners (1 vs. >1), and lifetime condom use (ever vs. never). The analysis was weighted using the HIV sample weights. Furthermore, for the two right-hand columns, the authors used a “3-level categorical variable reflecting 2 categories of lifetime physical IPV (ie, physical IPV only and physical IPV with forced sex) with no IPV as the referent group for all logistic analyses.” (p. 705) It is unclear from this text whether those experiencing only forced sex fall into the reference category or not. We have assumed here that they do.

2. This study included previously married women in all countries who answered the domestic violence module. It also excluded any individuals with missing information on any covariate, while Silverman et al. imputed values in 100 cases where covariate information was missing. In line with this, and to maintain a comparable sample size, we impute 594 individuals with missing information on condom use to have never used condoms.

3. The covariates used in this study included all those used by Silverman et al. (except condom use, which was not available in several countries) and additionally adjusted for marital status (current vs. previously married), urban residence, occupation (unemployed, manual, agricultural, other) and religious affiliation (Christian, Hindu Muslim, other).
